# Activin-dependent signaling in fibro/adipogenic progenitors causes fibrodysplasia ossificans progressiva

**DOI:** 10.1038/s41467-018-02872-2

**Published:** 2018-02-02

**Authors:** John B. Lees-Shepard, Masakazu Yamamoto, Arpita A. Biswas, Sean J. Stoessel, Sarah-Anne E. Nicholas, Cathy A. Cogswell, Parvathi M. Devarakonda, Michael J. Schneider, Samantha M. Cummins, Nicholas P. Legendre, Shoko Yamamoto, Vesa Kaartinen, Jeffrey W. Hunter, David J. Goldhamer

**Affiliations:** 1 Department of Molecular and Cell Biology, University of Connecticut Stem Cell Institute, University of Connecticut, Storrs, CT 06269 USA; 2 Department of Biological and Materials Sciences, School of Dentistry, University of Michigan, Ann Arbor, MI 48109 USA; 3 Alexion Pharmaceuticals, 100 College St, New Haven, CT 06510 USA

## Abstract

Fibrodysplasia ossificans progressiva (FOP) is a rare autosomal-dominant disorder characterized by progressive and profoundly disabling heterotopic ossification (HO). Here we show that fibro/adipogenic progenitors (FAPs) are a major cell-of-origin of HO in an accurate genetic mouse model of FOP (*Acvr1*^*tnR206H*^). Targeted expression of the disease-causing type I bone morphogenetic protein (BMP) receptor, ACVR1(R206H), to FAPs recapitulates the full spectrum of HO observed in FOP patients. ACVR1(R206H)-expressing FAPs, but not wild-type FAPs, activate osteogenic signaling in response to activin ligands. Conditional loss of the wild-type *Acvr1* allele dramatically exacerbates FAP-directed HO, suggesting that mutant and wild-type ACVR1 receptor complexes compete for activin ligands or type II BMP receptor binding partners. Finally, systemic inhibition of activin A completely blocks HO and restores wild-type-like behavior to transplanted *Acvr1*^*R206H/+*^ FAPs. Understanding the cells that drive HO may facilitate the development of cell-specific therapeutic approaches to inhibit catastrophic bone formation in FOP.

## Abstract (web summary)

Fibrodysplasia ossificans progressiva is a severe disorder characterized by heterotopic ossification, and is caused by mutations in ACVR1. Here, the authors show that expression of mutant ACVR1 in fibro/adipogenic progenitors recapitulates disease progression, and that this can be halted by systemic inhibition of activin A in mice.

## Introduction

Progressive ossification of skeletal muscle and associated soft tissues is the defining clinical manifestation of fibrodysplasia ossificans progressiva (FOP), a rare autosomal-dominant disorder caused by mutations in the type I BMP receptor, ACVR1 (ALK2)^1^. Episodic heterotopic ossification (HO) flares can occur spontaneously, without any known triggers, or can result from minor bumps and bruises, immunizations, dental work, and other mild soft tissue injuries. There are currently no effective treatments to prevent or limit the progression of HO, and surgical intervention to remove heterotopic bone in FOP patients is contraindicated because of the high risk of stimulating aggressive new bone growth^2^. Common cumulative effects of these HO flares, which typically begin in early childhood, include progressive immobility resulting from ankylosing joints, spinal fusions, diminished skeletal muscle function, and shortened life-span, most frequently due to thoracic insufficiency syndrome^3^.

Approximately 97% of FOP cases result from a single amino acid change (arginine to histidine at position 206; R206H) in the glycine–serine-rich (GS) domain of ACVR1^1,4^. Most studies have indicated that the mutant receptor is hypersensitive to BMP ligands^5,6^. Importantly, however, two recent reports demonstrated that the R206H substitution in ACVR1 is neomorphic, altering signaling specificity to activins^7,8^. Whereas activins normally activate SMAD 2/3 phosphorylation and function as inhibitors of BMP signaling^7–9^, activin binding to ACVR1(R206H)-containing complexes elicits an osteogenic response in competent cells through SMAD 1/5/8 phosphorylation^7,8^. Further, activins were shown to be obligatory ligands for HO in a genetically accurate mouse model of FOP^8^. While cumulative evidence indicates that proximate signaling events resulting from ligand-receptor engagement are shared by many cell types (e.g. phosphorylation of downstream effectors), transcriptional outputs and developmental consequences of this signaling are likely to be highly cell type-specific. As such, a mechanistic understanding of the link between receptor signaling and HO requires identification of the relevant offending cell populations in FOP.

Using lineage tracing and intramuscular injection of BMP2 as a mouse model for HO, we previously identified a progenitor population, lineage marked by expression of Tie2-Cre^10^, that is a major cell-of-origin for BMP2-induced endochondral bone formation^11,12^. Further analyses revealed that these so-called Tie2+ progenitors^12^ are resident in the skeletal muscle interstitium but distinct from endothelial and hematopoietic cells, exhibit multipotency, and are defined by expression of the cell surface markers PDGFRα, and SCA1^12^. Cumulative evidence has shown that Tie2+ progenitors are either identical to, or represent a subset of, fibro/adipogenic progenitors (FAPs)^13,14^, tissue-resident progenitors associated with pathogenic accumulation of fatty and fibrotic tissues in skeletal muscle^15–17^. Differences in cell surface marker expression and lack of myogenic capacity distinguishes FAPs from satellite cells, PW1+ interstitial cells, mesoangioblasts, muscle side population cells, and other known muscle-resident progenitors^18^.

We developed a conditional knockin model of FOP, in which expression of *Acvr1*^*R206H*^ from the endogenous *Acvr1* locus is dependent on Cre-mediated recombination. Using this accurate genetic model of FOP, we demonstrate that FAPs are a major offending cell type for both injury-induced and spontaneous HO, consistent with the recent study of Dey et al.^19^, who postulated that HO progenitors in FOP represent “reprogrammed” FAPs. Further, we show by lineage analysis that *Acvr1*^*R206H*^ functions cell-autonomously in FAP-directed endochondral bone formation. FAP-driven HO is strictly dependent on activin ligands and genetic analysis suggests that wild-type ACVR1 and ACVR1(R206H) compete for limiting ligand or essential signaling components, thereby dictating the extent of HO. Identifying the cells responsible for HO may facilitate development of cell-targeted therapeutic approaches for FOP, complementing ongoing drug development strategies based on inhibition of activin A^8,20^, ACVR1 kinase activity^19^ and cartilage differentiation^21–23^.

## Results

### Conditional activating *Acvr1* FOP allele

**Fig. 1:**
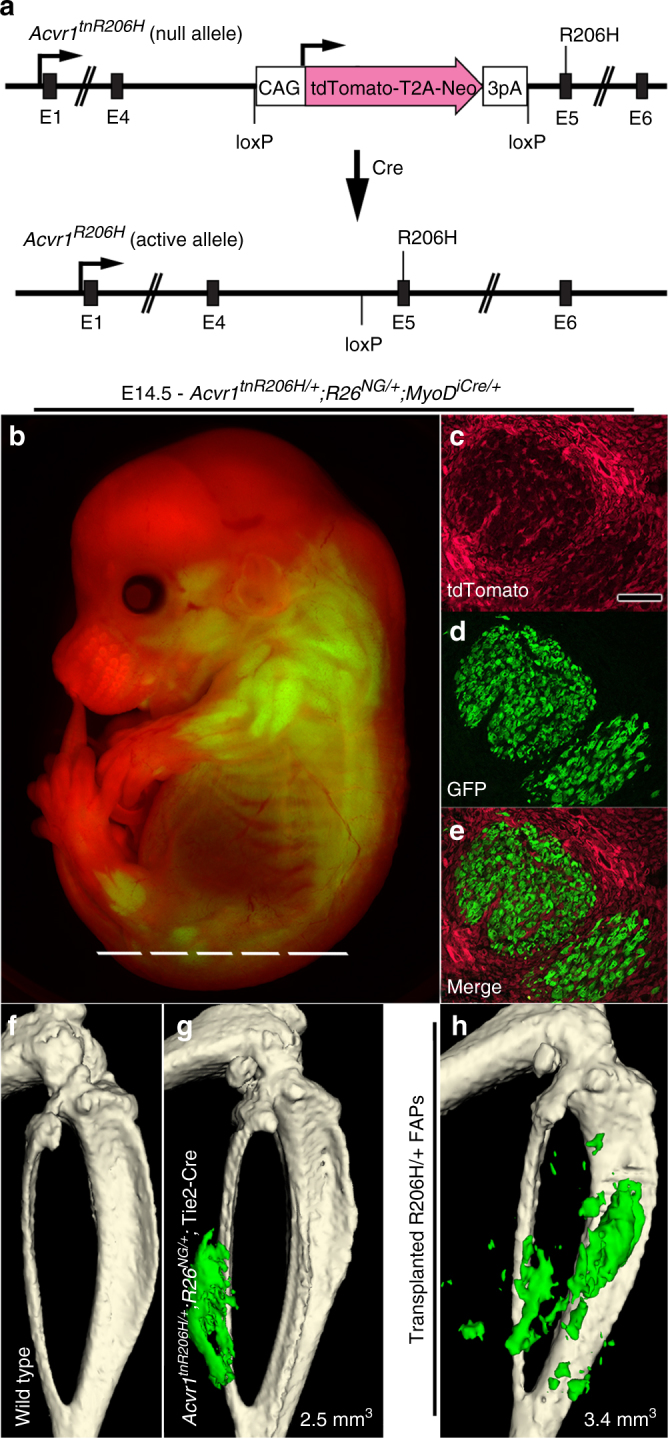
Targeted recombination of *Acvr1*^*tnR206H*^ in FAPs causes HO. **a** Organization of the conditional *Acvr1*^*tnR206H*^ knockin allele. **b**–**e** Validation of the fluorescence marking system using the *MyoD*^*iCre*^ driver. In *Acvr1*^*tnR206H/+*^;*R26*^*NG/+*^;*MyoD*^*iCre/+*^ mice, developing skeletal muscle expressed the GFP lineage tracer (*R26*^*NG*^), but not tdTomato. All other tissues express tdTomato but not GFP. **c**–**e** Cryosection through a developing hindlimb muscle bed. The approximate section plane is shown in **b**. **f**,**g** Representative µCT images of the distal hindlimb 14 days following pinch injury of the gastrocnemius muscle in wild-type (**f**; *n* = 25 mice) and *Acvr1*^*tnR206H/+*^;*R26*^*NG/+*^;Tie2-Cre (**g**; *n* = 42 mice) mice. **h** Representative µCT image of the distal hindlimb of a SCID host 21 days following intramuscular transplantation of *Acvr1*^*R206H/+*^ FAPs (*n* = 7 mice). HO in **g** and **h** is pseudocolored green and heterotopic bone volume is given (mm^3^)

We developed a conditional knockin mouse model of FOP in which expression of *Acvr1*^*R206H*^ is Cre-dependent and under regulatory control of the endogenous *Acvr1* locus (*Acvr1*^*tnR206H*^; Fig. 1a), enabling cell-specific and temporal control of *Acvr1*^*R206H*^ expression. This is related to a recently described FOP mouse model (*Acvr1*^*[R206H]FlEx*^)^8^, but includes the ability to track cells based on their recombination status in order to quantify recombination efficiency and to assess the cellular composition of lesional tissue. A floxed tdTomato reporter-stop cassette driven by the constitutively active CAG promoter/enhancer was inserted into intron 4 of the mouse *Acvr1* gene to serve the dual purpose of stopping transcription upstream of the FOP mutation in exon 5 (resulting in a presumptive null allele) in the absence of Cre, while allowing identification of unrecombined cells by red fluorescence. Cre-dependent excision of the CAG-tdTomato-stop cassette (*Acvr1*^*tnR206H/+*^ to *Acvr1*^*R206H/+*^) results in the loss of red fluorescence and read-through transcription of the R206H-encoding mutation. To test for Cre-dependent loss of red fluorescence, *Acvr1*^*tnR206H/+*^;*R26*^*NG/+*^ mice were crossed with mice carrying *MyoD*^*iCre*^, a well-characterized and efficient Cre driver specifically expressed in skeletal myoblasts and developing muscle fibers^24–26^. The Cre-dependent GFP reporter, *R26*^*NG*^, was included as a highly sensitive positive read-out of Cre activity^12,25^. Mouse embryos of the genotype *Acvr1*^*tnR206H/+*^;*R26*^*NG/+*^;*MyoD*^*iCre/+*^ showed widespread red fluorescence in non-muscle tissues (Fig. 1b, c, e), whereas maturing muscle fibers were GFP+ and lacked red fluorescence (Fig. 1d, e), confirming Cre-dependent loss of tdTomato expression.

### Expression of *Acvr1*^*R206H*^ in FAPs causes injury-induced HO

We used several Cre drivers with distinct specificities to target *Acvr1*^*R206H*^ expression to candidate cell types previously implicated in HO. Muscle stem cells (satellite cells) were targeted for *Acvr1*^*R206H*^ expression using the *MyoD*^*iCre*^ driver^24,25^. Pinch or cardiotoxin-mediated injury of the gastrocnemius or tibialis anterior hindlimb muscles of adult *Acvr1*^*tnR206H/+*^;*R26*^*NG/+*^;*MyoD*^*iCre*^ mice did not cause HO, as assessed by μCT or whole mount Alcian Blue/Alizarin Red (ABAR) staining, which allows simultaneous visualization of cartilage and bone (Supplementary Table 1). Similarly, directing *Acvr1*^*tnR206H*^ recombination to endothelial cells with VE-Cadherin-Cre^27^ did not result in HO following muscle pinch injury (Supplementary Table 1). Thus, despite the osteogenic activity of satellite cells/myoblasts^28–30^ and endothelium^31,32^ in certain experimental settings, expression of *Acvr1*^*R206H*^ in these cell types is not sufficient to induce HO in this genetic model of FOP.

To test whether FAPs represent a cell-of-origin for FOP, we directed recombination of *Acvr1*^*tnR206H*^ in FAPs using Tie2-Cre transgenic mice^10,12^. *Acvr1*^*tnR206H/+*^;*R26*^*NG/+*^;Tie2-Cre mice were recovered at Mendelian ratios at weaning (*n* = 111 mice scored), and adult mice were viable and fertile. Pinch injury of hindlimb skeletal muscle of *Acvr1*^*tnR206H/+*^;*R26*^*NG/+*^;Tie2-Cre adult mice resulted in HO in 100% of cases, whereas HO was not observed in mice lacking either *Acvr1*^*tnR206H*^ or Tie2-Cre (Fig. 1f, g; Supplementary Table 1). Heterotopic skeletal lesions, as analyzed by μCT or ABAR staining, showed that lesional tissue was typically embedded in muscle and associated soft tissues (Fig. 1g; Supplementary Fig. 1a, b), although close apposition or fusion with limb skeletal elements was sometimes observed (Supplementary Fig. 1c). Similar results were observed when skeletal muscle was injured by cardiotoxin injection, except that this less localized injury stimulus occasionally resulted in tendon/ligament HO (Supplementary Fig. 1c). Lineage analysis of *Acvr1*^*tnR206H/+*^;*R26*^*NG/+*^;Tie2-Cre mice confirmed the presence of Tie2+ cells in the Achilles tendon, and direct pinch injury of the Achilles tendon of these mice resulted in HO (Supplementary Fig. 1e-h).

Since Tie2-Cre is also expressed by endothelium and cells of the hematopoietic system^10,12^, we utilized two additional approaches to assess the FAP origin of HO lesions. First, we conducted complementary muscle injury studies with *Acvr1*^*tnR206H/+*^;*R26*^*NG/+*^ mice that carry the Pdgfrα-Cre transgene^33^. The cell surface receptor PDGFRα is the most specific single marker for muscle FAPs identified to date, and it is not expressed by endothelium^12,15^. In all cases, skeletal muscle exhibited robust, injury-induced HO (Supplementary Fig. 1d). Second, we developed a transplantation assay to test the osteogenic activity of *Acvr1*^*R206H*^-expressing FAPs and to verify that Tie2-lineage-labeled FAPs reside in native muscle tissue. GFP+ cells with the marker profile CD31-CD45-PDGFRα+ SCA1+ were isolated from total hindlimb muscles of *Acvr1*^*tnR206H/+*^;*R26*^*NG/+*^;Tie2-Cre mice by fluorescence-activated cell sorting (FACS) (Supplementary Fig. 2)^12,34^, and the recombined, tdTomato-negative FAP subfraction was expanded in culture. Intramuscular transplantation of ~ 10^6^ of these *Acvr1*^*R206H/+*^ FAPs into 1-day-preinjured SCID mice resulted in HO in the absence of exogenous ligand (Fig. 1h). In contrast, wild-type FAPs exhibited osteogenic activity only when transplanted in the presence of BMP2 (see below)^12^. Collectively, these data identify *Acvr1*^*R206H/+*^ FAPs as key drivers of HO. Further, the osteogenic activity of *Acvr1*^*R206H*/+^ FAPs following transplantation into SCID mice, which are wild-type for *Acvr1*, indicates that *Acvr1*^*R206H*^ expression in FAPs alone is sufficient for pathological bone formation.

### ACVR1 expression and inhibition of skeletal muscle regeneration

**Fig. 2:**
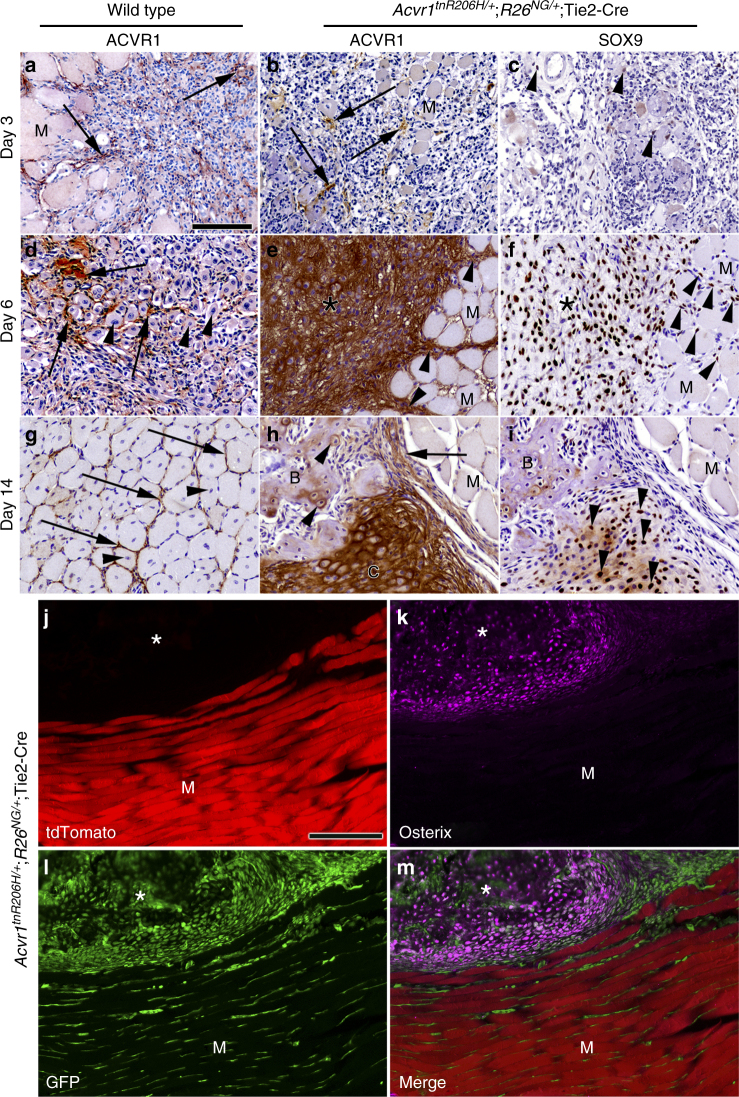
Histological and lineage analysis of FAP-driven HO. **a**–**i** IHC (brown staining) for ACVR1 and SOX9 in paraffin sections of pinch-injured gastrocnemius muscle in wild-type (*n* = 5 mice per timepoint) and FOP (*Acvr1*^*tnR206H/+*^;*R26*^*NG/+*^;Tie2-Cre; *n* = 5 mice per timepoint) mice. **a**–**c** At Day 3 post-injury, areas of weak ACVR1 staining (arrows) are evident in both wild-type (**a**) and FOP (**b**) muscle. Occasional SOX9+ cells were observed in FOP (**c**, arrowheads) and wild-type (see Supplementary Fig. 3) muscle. A few nascent muscle fibers (identified by their central nucleation; arrowheads) are present in wild-type muscle (**a**). **d**–**f** At Day 6, regenerating muscle fibers in wild-type muscle are (**d**, examples at arrowheads) surrounded by ACVR1+ interstitium (arrows). In FOP muscle, early cartilage and associated mesenchyme (asterisks) is intensely stained for ACVR1 (**e**) and SOX9 (**f**). Uninjured muscle adjacent to lesions exhibit thickened bands of interstitial cells that stain strongly for ACVR1 and SOX9 (arrowheads in **e**, **f**). **g**–**i** At Day 14, regenerated muscle fibers in wild-type muscle are surrounded by an ACVR1+ endomysial layer (**g**, arrows). In FOP muscle, cartilage (C) continued to strongly express ACVR1 (**h**, asterisk) and SOX9 (**i**, arrowheads). ACVR1 staining in some heterotopic bone cells (arrowheads) and encapsulating fibroblastic cells (arrow) is evident in **h**. M, uninjured muscle. Sections were counterstained with hematoxylin. **j**–**m** Fluorescence images of a cryosection through a heterotopic lesion (asterisks) 12 days after cardiotoxin injection (*n* = 7 mice). **j** Unrecombined, tdTomato+ cells were absent from skeletal lesional tissue, whereas muscle fibers (M) exhibited intense red fluorescence. **k** At this stage, most lesional tissue stained positively for the bone marker, osterix (purple). **l** Virtually all lesional cells are GFP+. **m** Merge of panels (**j**–**l**). Scale bars in **a**–**i** and **j**–**m** = 100 µm

Histological analyses showed that injury-induced heterotopic bone forms through an endochondral pathway in *Acvr1*^*tnR206H/+*^;*R26*^*NG/+*^;Tie2-Cre mice, with a developmental time course similar to that following intramuscular BMP2 injection into wild-type mice^12^. At 3 days post-injury, wild-type and *Acvr1*^*tnR206H/+*^;*R26*^*NG/+*^;Tie2-Cre muscle exhibited similar histological features, characterized by weak, diffuse, staining for ACVR1, increased cellularity (Fig. 2a, b), and sporadic expression of the chondrogenic marker, SOX9 (Fig. 2c; Supplementary Fig. 3a). SOX9 expression in wild-type muscle at this stage is consistent with a previous report of transient SOX9 expression during early myogenic differentiation^26^. By 6 days post-injury, however, the developmental trajectory of wild-type and *Acvr1*^*tnR206H/+*^;*R26*^*NG/+*^;Tie2-Cre muscle was dramatically divergent. Injured areas of wild-type muscle were filled with small, regenerated muscle fibers, identified by their central nucleation (Fig. 2d). These nascent fibers were surrounded by ACVR1-expressing mesenchymal cells and SOX9+ cells were rare (Fig. 2d; Supplementary Fig. 3b). In contrast, regenerated muscle fibers were rarely observed in *Acvr1*^*tnR206H/+*^;*R26*^*NG/+*^;Tie2-Cre muscle in areas of lesion formation. Instead, injured muscle contained large numbers of chondrocytes and accumulations of fibroblastic cells that intensely stained for SOX9 and ACVR1 (Fig. 2e, f). Intriguingly, thick bands of cells that stained strongly for ACVR1 and SOX9 were observed in uninjured areas adjacent to lesional tissue at this stage (Fig. 2e, f). We speculate that these cellular bands are areas of cell recruitment into lesional tissue, representing a mechanism of lesional growth. By day 14, regeneration of wild-type muscle was essentially complete, and regenerated muscle fibers were surrounded by a thin endomysial connective tissue layer that was often positive for ACVR1 (Fig. 2g). By day 14 in *Acvr1*^*tnR206H/+*^;*R26*^*NG/+*^;Tie2-Cre mice, most cartilage had been replaced by bone. The remaining cartilage stained positively for ACVR1, as did the fibrous tissue that tended to encapsulate lesions, whereas weak and sporadic ACVR1 staining was observed in emergent bone (Fig. 2h, i).

### *Acvr1*^*R206H*^ functions cell autonomously in FAPs

We sought to determine whether muscle injury drives *Acvr1*^*R206H/+*^ FAPs into both chondrogenic and osteogenic lineages and whether recombination at the *Acvr1*^*tnR206H*^ locus in FAPs is a requirement for skeletogenic lineage progression. Virtually all cells contributing to injury-induced skeletal tissue in *Acvr1*^*tnR206H*/+^;*R26*^*NG/+*^;Tie2-Cre muscle were lineage-labeled (GFP+) and the great majority of these were recombined at the *Acvr1*^*tnR206H*^ locus (1 osterix+ bone cell and no chondrocytes were tdTomato-positive; >500 of each scored) (Fig. 2j–m; Supplementary Fig. 4a–d), indicating that *Acvr1*^*R206H/+*^ FAPs represent the predominant cell-of-origin for both heterotopic cartilage and bone in this FOP model. A priori, the scarcity of tdTomato-positive skeletal lesional cells could result from an exceptionally high Cre recombination efficiency at the *Acvr1*^*tnR206H*^ locus such that unrecombined FAPs were rare in muscle tissue. FACS analysis of FAPs from *Acvr1*^*tnR206H*/+^; *R26*^*NG/+*^;Tie2-Cre muscle, however, showed that Tie2-Cre-dependent recombination at the *Acvr1*^*tnR206H*^ locus is rather inefficient; approximately 10 and 20% of FAPs were recombined at day 0 and at 3 days post-injury, respectively (Supplementary Fig. 2c, d). Further, the lack of tdTomato-positive cells in definitive heterotopic cartilage or bone was not due to inadequate detection sensitivity, as unrecombined, tdTomato-positive cells were readily detected in heterotopic skeletal lesions following intramuscular injection of BMP2 (Supplementary Fig. 4g, j). Accordingly, virtually all BMP2-induced lesional cartilage and bone was tdTomato-positive when recombination at the *Acvr1*^*tnR206H*^ locus was induced by *MyoD*^*iCre*^ or VE-Cadherin-Cre drivers (Supplementary Fig. 4e, f, h, i, k-o), further demonstrating that *Acvr1*^*R206H*^-expressing satellite cells and endothelium do not exhibit osteogenic activity, even under conditions of excessive BMP2 stimulation.

We also addressed cell autonomy in the transplantation model. GFP + tdTomato- *Acvr1*^*R206H/+*^ FAPs from *Acvr1*^*tnR206H*/+^;*R26*^*NG/+*^;Tie2-Cre muscle were transplanted into the preinjured gastrocnemius muscle of SCID mice. Histological analyses at 21 days post-transplantation revealed that the vast majority of heterotopic cartilage and bone was derived from transplanted cells. Unlabeled cells (presumptive host cells) were associated with lesional tissue, but only infrequently (~5%) contributed to definitive cartilage or bone at this stage (Supplementary Fig. 5a, b). Transplanted wild-type FAPs did not undergo skeletogenesis in the absence of exogenously added BMP2, as shown previously^12^ and, instead, surrounded regenerated muscle fibers and occasionally gave rise to fibrotic tissue (Supplementary Fig. 5c). Collectively, these data indicate that *Acvr1*^*R206H*^ expression in FAPs is necessary for initiation of FAP-directed endochondral bone formation and that *Acvr1*^*R206H*^-non-expressing host cells only occasionally contribute to HO at the stages examined.

### FAPs drive spontaneous HO

**Fig. 3:**
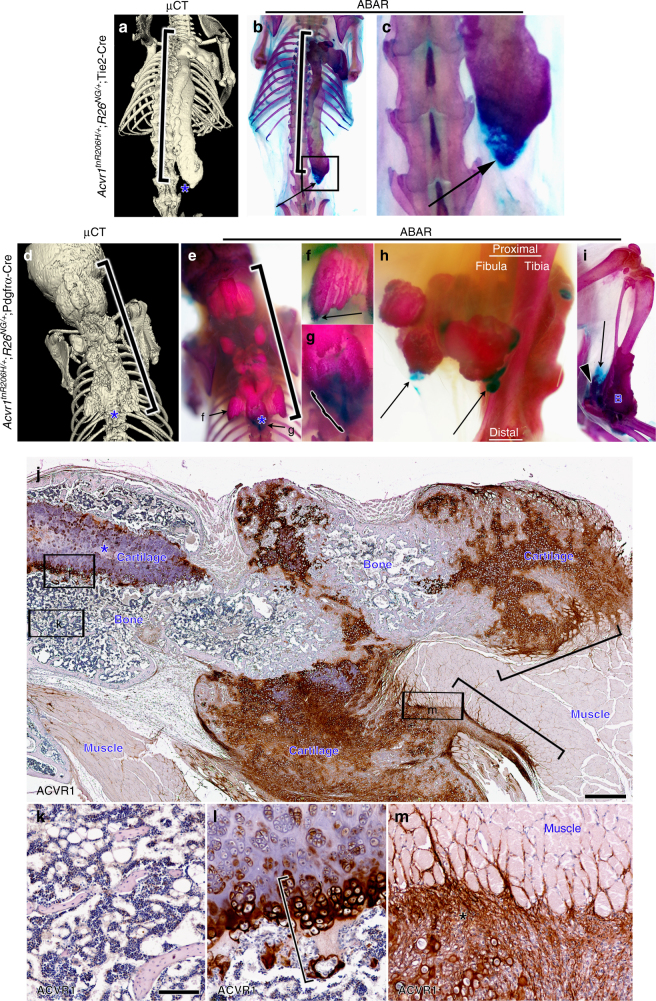
FAPs drive spontaneous HO via endochondral ossification. **a**–**c** Spontaneous HO extended along the spine of a 22-week-old *Acvr1*^*tnR206H/+*^;*R26*^*NG/+*^;Tie2-Cre mouse. **a** µCT detects calcified heterotopic bone (bracket) but not the distal cartilaginous region of the lesion (asterisk). **b** Whole mount ABAR staining of the same mouse detects both lesional cartilage (blue; arrow) and bone (red; bracket). **c** Magnified image of the boxed region in **b**. **d–g** Spontaneous HO (bracket) along the spine of a 6-week-old *Acvr1*^*tnR206H/+*^;*R26*^*NG/+*^;Pdgfrα-Cre mouse. The site of the most posterior chondrogenic area is shown at the asterisk in **d** and **e**. **e** ABAR staining shows the presence of two chondrogenic areas at the periphery of the predominantly boney lesion. **f**, **g** Higher magnification images of areas denoted in **e**. Chondrogenic areas are shown at the arrow or bracket. **h**,**i** ABAR staining of the distal hindlimb of two 6-week-old *Acvr1*^*tnR206H/+*^;*R26*^*NG/+*^;Pdgfrα-Cre mice. Multiple chondrogenic areas (examples at arrows) are evident at the periphery of intramuscular boney lesions (**h**). In **i**, peripheral cartilage (arrow) is associated with HO of the Achilles tendon (arrowhead). B, heterotopic bone associated with the distal tibia/fibula. **j**–**m** Paraffin section of spontaneous HO from an adult *Acvr1*^*tnR206H/+*^;*R26*^*NG/+*^;Pdgfrα-Cre mouse stained by IHC for ACVR1 (brown) and counterstained with hematoxylin (*n* = 7 mice). **j** Low magnification image showing multiple areas of heterotopic cartilage and bone. Areas of mature (asterisk) and immature cartilage are evident. Staining for ACVR1 was most intense in immature cartilage, peripheral regions of mature cartilage, and in fibroproliferative and chondrogenic regions at the junction between HO and muscle (brackets). **k**–**m** Higher magnification images of the labeled boxes in **j**. **k** ACVR1 expression was not detected in apparently mature heterotopic bone, characterized by abundant marrow elements. **l** Growth plate-like cartilaginous regions strongly stain for ACVR1 at the chondro-osseous junction (bracket). **m** Peripheral fibroproliferative and chondrogenic regions (asterisk) strongly expressed ACVR1. Scale bars = 500 µm (**j**) and 100 µm (**k**–**m**)

HO flares often occur in the absence of known inflammatory or injury triggers, particularly in young FOP patients, and this mode of HO, often referred to as spontaneous HO, is a major contributor to morbidity^25^. To test whether FAPs also represent a major cell-of-origin for spontaneous HO, we aged a cohort of singly-housed, *Acvr1*^*tnR206H/+*^;*R26*^*NG/+*^;Tie2-Cre mice, and visualized HO by μCT and ABAR staining. HO was observed in 12 of 15 mice by 1 year-of-age (Fig. 3a–c; Supplementary Table 1). 5.5 months represented the earliest age of HO detection in this small cohort.

Targeting recombination with the Pdgfrα-Cre driver resulted in earlier-onset and more widely distributed disease, characterized by HO of the musculature, tendons, and ligaments at diverse anatomical locations. HO was infrequently noted in some *Acvr1*^*tnR206H/+*^;*R26*^*NG/+*^;Pdgfrα-Cre mice by 2-weeks-of-age, was present in all 4-week-old mice, and was extensive in all surviving mice by 6-weeks-of-age (Fig. 3d–i). While a full characterization of *Acvr1*^*tnR206H/+*^;*R26*^*NG/+*^;Pdgfrα-Cre mice will be reported elsewhere, we note here a substantially higher recombination efficiency at the *Acvr1*^*tnR206H*^ locus in FAPs carrying Pdgfrα-Cre (~40%) compared to Tie2-Cre (~10%) (Supplementary Fig. 2c). Additionally, lineage tracing revealed labeling of periosteum with the Pdgfrα-Cre driver, but not the Tie2-Cre driver (Supplementary Fig. 6). These factors likely explain, at least in part, the earlier onset and greater severity of disease in *Acvr1*^*tnR206H/+*^;*R26*^*NG/+*^;Pdgfrα-Cre mice.

Injury-induced and spontaneous HO share mechanistic similarities in addition to a common origin of skeletogenic progenitors. Like injury-induced HO, we found that skeletal elements resulting from spontaneous HO are derived almost exclusively from *Acvr1*^*R206H*^-recombined cells (Supplementary Fig. 7). Further, spontaneous lesions in both *Acvr1*^*tnR206H/+*^;*R26*^*NG/+*^;Pdgfrα-Cre and *Acvr1*^*tnR206H/+*^;*R26*^*NG/+*^;Tie2-Cre mice developed by an endochondral pathway, as evidenced by the presence of chondrogenic regions in most lesions (Fig. 3a–i). Interestingly, we note that these chondrogenic regions, which were typically at the periphery of lesional tissue (Fig. 3e–i), exhibited robust ACVR1 expression (Fig. 3j). ACVR1 expression was not detected in established lesional bone (Fig. 3k), but was robust at chondro-osseous junctions (Fig. 3l) and in the muscle interstitium adjacent to lesional tissue, which morphologically resembled injury-induced HO (compare Fig. 3m to Fig. 2e). We speculate that these chondrogenic and interstitial cell regions constitute sites of directional lesional growth, which could be driven by chondrocyte proliferation, as for growth of long bones at the growth plates, or by continual recruitment of FAPs into the chondrogenic lineage.

### Activins induce osteogenic differentiation of *Acvr1*^*R206H/+*^ FAPs

**Fig. 4:**
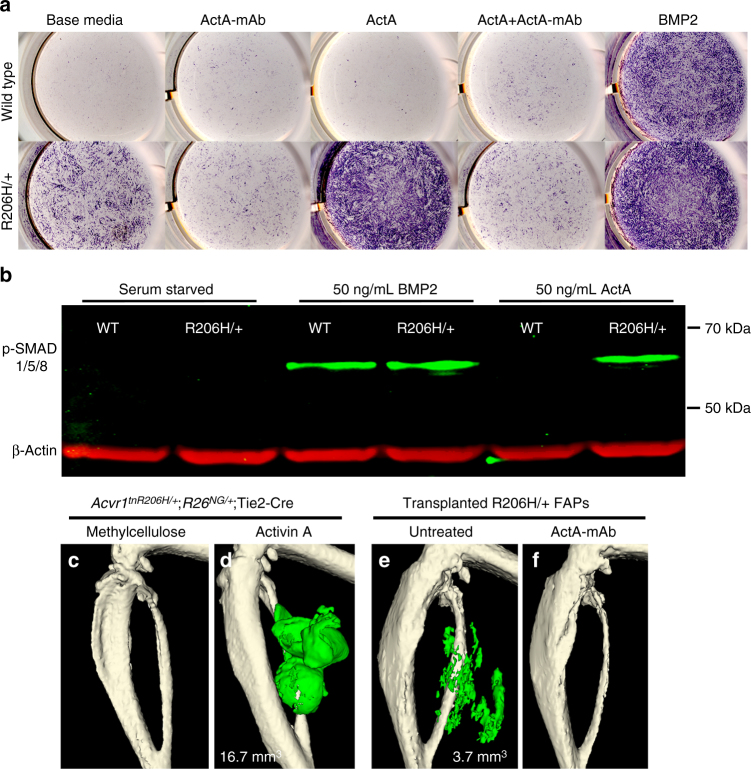
Activin A is an osteogenic ligand for *Acvr1*^*R206H/+*^ FAPs. **a** Osteogenic differentiation of *Acvr1*^*R206H/+*^ FAPs (R206H/+; *n* = 6 mice) and wild-type FAPs (*n* = 6 mice) cultured in base media (5% FBS/DMEM), with or without 25 ng/mL (~1 nM) activin A or BMP2, was assessed by ALP staining (purple). ActA-mAb was used at 1 μg/mL (7-fold molar excess over ligands). **b** Western blot of SMAD 1/5/8 phosphorylation (p-SMAD 1/5/8) in response to 50 ng/mL BMP2 or 50 ng/mL activin A. β-actin was used as a loading control (*n* = 3 mice per genotype). **c**, **d** µCT of the distal hindlimb of *Acvr1*^*tnR206H/+*^;*R26*^*NG/+*^;Tie2-Cre mice 14 days post-injection of 1% methylcellulose carrier alone (**c**; *n* = 8 mice) or 5 µg activin A in methylcellulose (**d**; *n* = 3 mice). **e**, **f** µCT of the distal hindlimb at day 21 post-transplantation of *Acvr1*^*R206H/+*^ FAPs (R206H/+; *n* = 8 mice) into the gastrocnemius of SCID hosts without **e** or with **f** a single dose of ActA-mAb administered to the SCID host at the time of muscle injury (1 day prior to transplantation). HO in **d** and **e** is pseudocolored green and heterotopic bone volume is given (mm^3^)

Recent studies have demonstrated that the R206H amino acid substitution in the GS domain of ACVR1 alters the signaling properties of the receptor such that activin binding activates BMP signaling and elicits an osteogenic response in competent cells^7,8^. To assess signaling properties of activins on cells demonstrably relevant to FOP, recombined (*Acvr1*^*R206H/+*^) FAPs from *Acvr1*^*tnR206H/+*^;*R26*^*NG/+*^;Tie2-Cre mice were FACS-isolated, expanded in culture, and tested for responsiveness to activins and BMP2. Using alkaline phosphatase (ALP) expression as a marker of osteogenic differentiation, we found that serum-containing medium without exogenously added ligand elicited a preferential osteogenic response in *Acvr1*^*R206H/+*^ FAPs relative to wild-type FAPs (Fig. 4a). Addition of 25 ng/mL activin A (~1 nM of β_A_β_A_ dimers) to the culture medium dramatically increased ALP staining of *Acvr1*^*R206H/+*^ FAPs, whereas wild-type FAPs were unresponsive to activin A (Fig. 4a). In contrast, wild-type and *Acvr1*^*R206H/+*^ FAPs showed comparable responsiveness to BMP2 at ligand concentrations ≥25 ng/mL (~1 nM BMP2 dimers) (Fig. 4a, b). Consistent with these findings, activin A stimulated phosphorylation of SMAD 1/5/8—key downstream mediators of canonical BMP signaling—in *Acvr1*^*R206H/+*^ FAPs, but not wild-type FAPs (Fig. 4b). Stimulatory effects of serum and exogenous activin A on *Acvr1*^*R206H/+*^ FAPs were reduced or eliminated by inclusion in the culture medium of a 7-fold molar excess of a blocking monoclonal antibody to activin A (ActA-mAb, kindly provided by Acceleron Pharma) (Fig. 4a). Activin B (β_B_β_B_ homodimer) stimulated a comparable level of ALP staining and SMAD 1/5/8 phosphorylation, but was not inhibited by ActA-mAb (Supplementary Fig. 8).

We next tested whether activin A was sufficient to induce FAP-driven HO in vivo. Five micrograms of activin A in a 1% methylcellulose carrier was injected into the gastrocnemius muscle and HO was assessed by μCT at 3 weeks after injection. Activin A produced a robust osteogenic response when injected into *Acvr1*^*tnR206H/+*^;*R26*^*NG/+*^;Tie2-Cre muscle (Fig. 4d), but not when injected into wild-type muscle. Injection of the methylcellulose carrier alone did not elicit HO in *Acvr1*^*tnR206H/+*^;*R26*^*NG/+*^;Tie2-Cre mice (Fig. 4c), demonstrating that the minor injury associated with intramuscular methylcellulose injection is insufficient to induce HO in this model. Interestingly, *Acvr1*^*tnR206H/+*^;*R26*^*NG/+*^;Tie2-Cre mice exhibited a mild osteogenic response to methylcellulose injection when 5 μg of activin A/methylcellulose was simultaneously injected into the contralateral leg (Supplementary Fig. 9), most likely by increasing systemic activin A levels and thereby reducing the injury/inflammation threshold required to induce a FAP-dependent osteogenic response. These data support and extend previous findings of activin A-induced HO in a distinct FOP model^8^, implicating FAPs as a key cellular target of activin A in vivo.

### Activins are obligatory ligands for FAP-driven HO

Previous studies have demonstrated that activins are obligatory ligands for HO in mice that were globally recombined at the *Acvr1*^*[R206H]FlEx*^ locus^8^. We tested whether ActA-mAb could inhibit FAP-directed HO in transplantation, injury-induced, and spontaneous FOP models. In the FAP transplantation model, administration of a single, 10 mg/kg dose of ActA-mAb to SCID hosts on the day of injury, 1 day prior to transplantation, prevented HO of transplanted FAPs (Fig. 4e, f). Engrafted FAPs of ActA-mAb-treated hosts instead assumed an apparently typical position in the endomysium surrounding host regenerated muscle fibers or, occasionally, formed aggregates of fibrotic tissue, similar to the behavior of transplanted wild-type FAPs (Supplementary Fig. 5c–e). Thus, pathogenic commitment of transplanted *Acvr1*^*R206H/+*^ FAPs to endochondral ossification is entirely dependent on activins ligands.

**Fig. 5:**
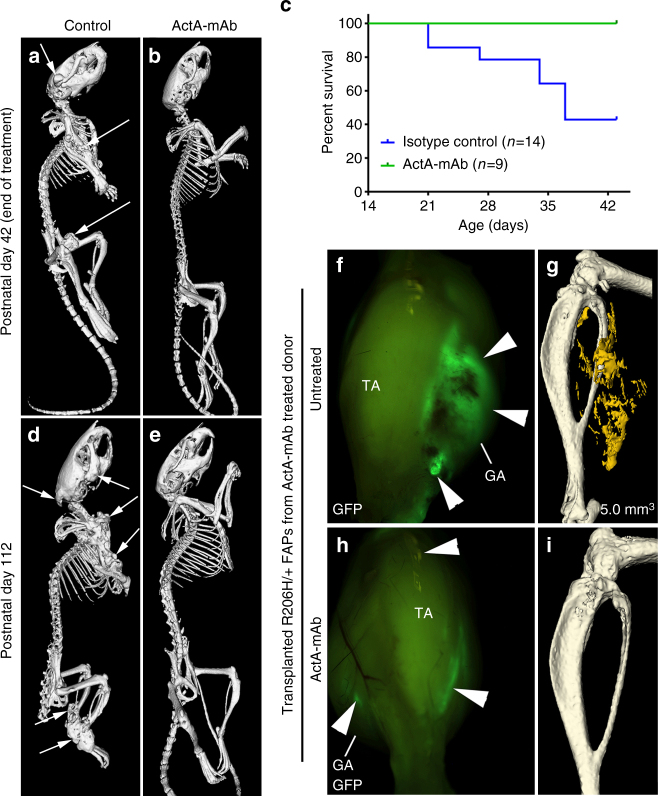
Activin blockade inhibits FAP-mediated spontaneous HO. **a**,** b** Representative µCT images of 6-week-old *Acvr1*^*tnR206H/+*^;*R26*^*NG/+*^;Pdgfrα-Cre mice treated with 10 mg/kg IgG2a isotype control antibody (**a**) or ActA-mAb (**b**) twice weekly from 14–42 days-of-age. Arrows denote sites of HO. **c** Survival curve of isotype control (*n* = 14) and ActA-mAb-treated (*n* = 9) mice. Percent survival reflects animal deaths as well as mice euthanized due to weight loss of >20%. **d**, **e** Representative µCT images of 112-day-old *Acvr1*^*tnR206H/+*^;*R26*^*NG/+*^;Pdgfrα-Cre mice either untreated (**d**; *n* = 1) and heavily burdened with HO of the musculature, tendons/ligaments and jaw (arrows), or 70 days post-cessation of ActA-mAb treatment (**e**; *n* = 6) and presenting no overt HO. **f**–**i** GFP-labeled *Acvr1*^*R206H/+*^ FAPs were isolated and expanded from 42-day-old *Acvr1*^*tnR206H/+*^;*R26*^*NG/+*^;Pdgfrα-Cre mice (*n* = 3) that had been treated with ActA-mAb from 14–42 days-of-age, and transplanted into the distal hindlimb of SCID hosts that were either untreated (**f**, **g**) (*n* = 6) or administered ActA-mAb on the day of injury (**h**,** i**) (*n* = 6). **f**,** h** Whole mount GFP images showing FAP engraftment (examples at arrowheads). **g**,** i** µCT images at 21 days post-transplantation. HO was only observed in untreated hosts. TA tibialis anterior, GA gastrocnemius. HO in **g** is pseudocolored yellow. Heterotopic bone volume is given (mm^3^)

We next addressed whether spontaneous, FAP-driven HO is strictly dependent on activin ligands. *Acvr1*^*tnR206H/+*^;*R26*^*NG/+*^;Pdgfrα-Cre mice were used for these experiments because of the early onset and consistency of disease progression over a relatively short temporal window. Beginning at 2 weeks-of-age, prior to the onset of HO, experimental mice received 10 mg/kg ActA-mAb twice weekly for 4 weeks and HO was assessed at 6 weeks-of-age by μCT. All control mice (*Acvr1*^*tnR206H*/+^;*R26*^*NG/+*^;Pdgfrα-Cre mice either untreated (*n* = 10) or injected with an isotype-matched IgG2a control antibody (*n* = 14)) that survived to 6-weeks-of-age exhibited extensive HO at multiple anatomical locations (Fig. 5a). Forty-three percent of control mice died or experienced >20% weight loss and were removed from the study prior to the 6-week-old endpoint (Fig. 5c). Jaw ossification, which was observed in all control mice, appeared to be the major cause of morbidity and mortality (Fig. 5a, c). In striking contrast, all mice treated with ActA-mAb survived to 6-weeks-of-age, and eight of nine mice showed no evidence of HO (Fig. 5b, c).

Unexpectedly, activin A inhibition provided long-term protection from spontaneous HO in *Acvr1*^*tnR206H*/+^;*R26*^*NG/+*^*;*Pdgfrα-Cre mice. For these experiments, mice were treated with ActA-mAb from 2 to 6-weeks-of-age, as above, and then allowed to age for an additional 10 weeks without further treatment. While the single surviving 16-week-old control mouse was heavily burdened with HO, spontaneous HO was not observed in 16-week-old mice that had previously received ActA-mAb (Fig. 5d, e). In addition, *Acvr1*^*R206H/+*^ FAPs from 6-week-old ActA mAb-treated *Acvr1*^*tnR206H*/+^;*R26*^*NG/+*^;Pdgfrα-Cre mice retained robust, activin-dependent, osteogenic activity following transplantation and in cell culture (Fig. 5f–i; Supplementary Fig. 10a, b), demonstrating that the intrinsic osteogenic capacity of *Acvr1*^*R206H/+*^ FAPs was not irreversibly altered by ActA-mAb treatment.

**Fig. 6:**
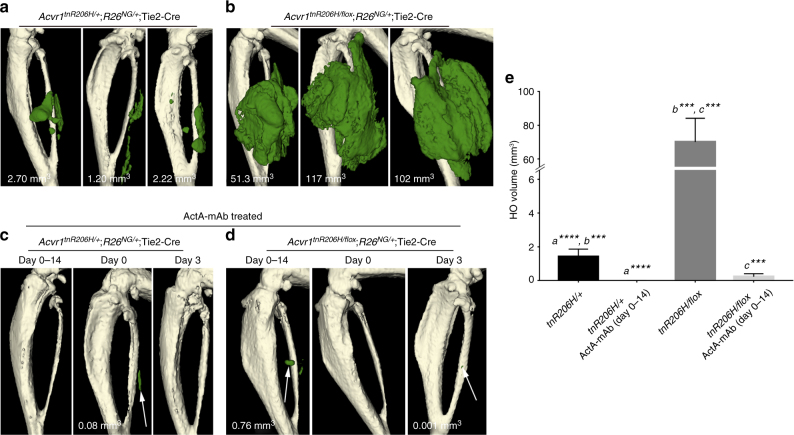
HO is greatly exacerbated by loss of wild-type *Acvr1*. **a**,** b** Representative µCT images of HO (pseudocolored green) 14 days after pinch injury of the gastrocnemius muscle of *Acvr1*^*tnR206H/+*^;*R26*^*NG/+*^;Tie2-Cre (**a**; *n* = 7) or *Acvr1*^*tnR206H/flox*^;*R26*^*NG/+*^;Tie2-Cre (**b**; *n* = 8) mice. **c**, **d** ActA-mAb administered at 10 mg/kg in both mouse genetic models, either twice weekly beginning at the time of injury (Day 0–14; tnR206H/+, *n* = 12; tnR206H/flox, *n* = 7), as a single dose at the time of injury (Day 0; tnR206H/+, *n* = 8; tnR206H/flox, *n* = 4), or as a single dose 3 days post-injury (Day 3; tnR206H/+, *n* = 12; tnR206H/flox, *n* = 4), was highly effective at inhibiting HO. Residual HO in these treated mice is shown at arrows. HO volume (mm^3^) in **a**–**d** is given. **e** Quantification of HO volume at day 14 post-injury from untreated and ActA-mAb-treated mice. Bars represent ± SEM. ***p < 0.001, ****p < 0.0001

Injury-induced HO was also effectively blocked when the same ActA-mAb dose was administered twice-weekly to *Acvr1*^*tnR206H*/+^;*R26*^*NG/+*^;Tie2-Cre mice, beginning on the day of injury (Fig. 6a, c). Further, a single ActA-mAb injection at the time of injury also provided near complete protection against HO development (Fig. 6c). Consistent with recent findings^20^, delaying antibody administration until day 3 post-pinch injury, when fibroproliferative expansion is approaching its maximal extent, but prior to the appearance of cartilage, was effective in inhibiting HO (Fig. 6c). Cartilage was not detected by ABAR staining in limbs lacking HO, indicating that the blockade in endochondral ossification occurred prior to cartilage development. Collectively, these data demonstrate that transduction of osteogenic signals by *Acvr1*^*R206H/+*^ FAPs is strictly ligand dependent and, given the specificity of ActA-mAb, suggest a fundamental role for activin A (and perhaps other β_A_-containing activins), in FOP pathogenesis in vivo.

### Wild-type ACVR1 dampens the HO response of *Acvr1*^*R206H*/+^ FAPs

As FOP patients are heterozygous for the *Acvr1*^*R206H*^ mutation, and wild-type and ACVR1(R206H) receptors bind activins^7–9^ but exhibit distinct ligand-dependent signaling properties, we investigated the effect of genetic loss of wild-type *Acvr1* to better understand the regulatory relationship between the wild-type and mutant receptors in FAP-directed HO. Thus, we quantified injury-induced HO in *Acvr1*^*tnR206H*^ mice that carried a conditional null allele of *Acvr1* (referred to here as *Acvr1*^*flox*^)^35,36^, *R26*^*NG*^, and Tie2-Cre (*Acvr1*^*tnR206H/flox*^;*R26*^*NG*/+^;Tie2-Cre). *Acvr1*^*tnR206H/flox*^;*R26*^*NG*/+^;Tie2-Cre mice were generated at expected Mendelian ratios (*n* = 94), and were viable and fertile. Loss of the wild-type *Acvr1* allele in these FOP mice resulted in a profound, 50-fold increase in HO volume (70.04 ± 14.05 mm^3^; SEM) compared to *Acvr1*^*tnR206H/+*^;*R26*^*NG/+*^;Tie2-Cre mice (1.41 ± 0.46 mm^3^; SEM) following pinch injury of the gastrocnemius muscle (Fig. 6b, e). These data are consistent with the hypothesis that the severity of FAP-directed HO is determined by competition between wild-type and mutant ACVR1 receptors for limiting quantities of type II BMP receptor binding partners or activin ligands. Notably, activin A inhibition with ActA-mAb almost completely blocked HO resulting from pinch injury of *Acvr1*^*tnR206H/flox*^;*R26*^*NG*/+^;Tie2-Cre muscle, as determined by μCT (Fig. 6d, e), demonstrating that even the explosive HO resulting from loss of wild-type ACVR1 remains strictly ligand dependent.

## Discussion

Since the pioneering work of Urist^37^ demonstrated the osteoinducing properties of demineralized bone matrix, the identity of tissue-resident cells responsible for HO has been vigorously debated^38^. Previous studies have largely used cell culture and in vivo models of HO that rely on exposure of wild-type cells to non-physiological levels of osteogenic BMPs, or over-expression of ACVR1(R206H) or ACVR1(Q207D)^30^, a constitutively active, ligand-independent engineered receptor that has not been observed in the FOP patient population^11,12,31,38–42^. Given the non-physiological conditions employed, the relevance of the candidate cell populations to FOP pathogenesis could not be critically evaluated. In the present report, we developed a physiologically relevant conditional expression allele of *Acvr1*^*R206H*^ and used Cre/lox lineage tracing methods to interrogate HO progenitors in FOP. We demonstrated that FAPs^13^ are a major contributor to both heterotopic cartilage and bone. We further showed that FAPs mediate both injury-induced and spontaneous disease, and cause HO in essentially all major anatomical sites described for FOP patients, including appendicular and back musculature, tendons/ligaments, major joints, and jaw. While the present experiments do not address whether FAPs are the only progenitor population with the capacity to drive HO, FAPs can explain the full repertoire of HO in FOP.

Using Cre/lox lineage-tracing methods similar to those of the present study, Dey et al. concluded that two distinct, non-overlapping tissue-resident progenitor cell populations are responsible for the diverse HO phenotypes in FOP^19^. This conclusion was based on the distinct HO phenotypes observed when expression of an *Acvr1*^*R206H*^ allele (*Acvr1*^*[R206H]FlEx*^)^8^ and an Acvr1(Q207D) transgene^30^ were directed by Scleraxis-Cre (Scx-Cre) and Mx1-Cre drivers, which mark spatially distinct populations. Thus, the Scx-Cre models were characterized by spontaneous HO lesions of tendons, ligaments and joints, whereas Mx1-Cre mice were susceptible to injury-dependent HO of skeletal muscle (neither model exhibited spontaneous HO of skeletal muscle)^19^. Notably, however, PDGFRα+ cells were shown to be a small subfraction of each lineage-labeled cell pool, and the PDGFRα+ subfractions exhibited significantly increased chondrogenic and osteogenic activity in culture compared to PDGFRα-negative subfractions^19^. As PDGFRα is the single best marker for FAPs^12,14^, a parsimonious explanation that reconciles these data with the present study is that FAPs are at least partially responsible for HO in the Scx-Cre and Mx1-Cre models, as postulated by Dey et al. for the Mx1+ lineage^19^, and that Scx-Cre and Mx1-Cre drivers define distinct spatial expression domains rather than identifying distinct progenitors of HO. In this regard, possible differences in recombination efficiency could have contributed to the Cre-specific phenotypes reported; we have observed a correlation between recombination efficiency of the *Acvr1*^*tnR206H*^ allele and the penetrance, severity, and anatomical location of spontaneous HO.

Understanding FOP pathogenesis will require detailed knowledge of the cell populations that drive the initiation, growth, and remodeling of HO. Although it is clear that the initiation of HO is driven by *Acvr1*^*R206H*/+^ progenitors, there is some disagreement concerning the degree to which wild-type cells can contribute to skeletal tissues of HO lesions. Dey et al.^19^ reported that Scx-Cre and Mx1-Cre lineage-labeled populations gave rise to heterotopic cartilage, but not heterotopic bone, implying cell non-autonomous functions of the mutant receptor. In contrast, using an *Acvr1*^*R206H*^ allele (*Acvr1*^*tnR206H*^) that allows its recombination status to be assessed in histological sections, we showed that *Acvr1*^*R206H*/+^ FAPs comprised the great majority of injury-induced heterotopic cartilage and bone, and *Acvr1*^*tnR206H*^-unrecombined cells only rarely (<1%) contributed to definitive skeletal tissue of HO lesions at the stages examined. Similarly, following transplantation of *Acvr1*^*R206H/+*^ FAPs into SCID muscle, apparent host cells accounted for only a small percentage (~5%) of definitive cartilage and bone. Chakkalakal et al.^43^, however, observed a much greater contribution (~35%) of wild-type cells to skeletal lesional tissues following muscle injury of mice chimeric for a constitutive *Acvr1*^*R206H*^ knockin allele. Although substantial differences in experimental approaches preclude direct comparisons between these studies, collectively, available data indicate some capacity of *Acvr1*^*R206H*^-non-expressing cells to contribute to heterotopic skeletal lesions. As wild-type FAPs, and possibly other cell types, can undergo BMP-induced endochondral bone formation in mouse models^12,38^, it is reasonable to propose that maturing heterotopic bone provides a signaling environment that is sufficiently BMP-rich to recruit *Acvr1*^*R206H*^-non-expressing cells to participate in bone growth and remodeling.

Activin A inhibition was shown to be highly effective at blocking HO in globally recombined *Acvr1*^*[R206H]FlEx*^ mice^8^. Consistent with these findings, we showed that HO mediated by *Acvr1*^*R206H/+*^ FAPs was strictly dependent on activin ligands; a monoclonal antibody directed against activin A inhibited HO in spontaneous, injury-induced, and transplantation models of FOP. Notably, even a single administration of ActA-mAb at the time of injury completely blocked injury-induced HO and osteogenic differentiation of transplanted *Acvr1*^*R206H/+*^ FAPs. The first few days after muscle injury are characterized by a 9-fold increase in local activin A levels^44^, a ~2- to 5-fold increase in FAP numbers^12,13^ (present study), a dramatic increase in ACVR1 protein levels (present study), and muscle inflammation—a well-known trigger for HO flares^34^. These observations raise the intriguing possibility that therapeutic intervention over a relatively narrow developmental and temporal window relative to initiation of an HO flare might be sufficient to block skeletogenic programming of *Acvr1*^*R206H/+*^ FAPs, thereby inhibiting HO.

ActA-mAb provided unexpectedly long protection against spontaneous HO. Thus, *Acvr1*^*tnR206H/+*^;*R26*^*NG/+*^;Pdgfrα-Cre mice treated with ActA-mAb from 2 to 6-weeks-of-age were free from HO through 16-weeks-of-age. Given the conclusion that MSCs treated with RAR-γ agonists lose skeletogenic potential and are thereby protected from subsequent BMP2-induced osteogenesis^22^, we tested the developmental capacity of *Acvr1*^*R206H/+*^ FAPs after prolonged ActA-mAb treatment. Importantly, *Acvr1*^*R206H/+*^ FAPs derived from *Acvr1*^*tnR206H/+*^;*R26*^*NG/+*^;Pdgfrα-Cre mice retained robust osteogenic potential, as demonstrated both in cell culture assays and following transplantation. These data demonstrate that prolonged inhibition of activins does not alter the osteogenic potential of *Acvr1*^*R206H/+*^ FAPs, and suggest that protection through 16-weeks-of-age is conferred by the persistence of sufficient antibody titers during the 10-week wash-out period to block the effects of circulating or tissue-derived activin A.

The profound increase in injury-induced HO in FOP mice when the wild-type *Acvr1* allele was targeted for deletion suggests that the wild-type ACVR1 protein serves as a direct or indirect inhibitor of ACVR1(R206H) signaling. Importantly, we showed that this exacerbated HO is also strictly dependent on activin ligands. As receptor complexes containing wild-type ACVR1 bind activins^7–9^ but are inactive in osteogenic signaling^7,8^, these data suggest a model in which wild-type ACVR1-containing activin-binding complexes serve as competitive inhibitors and dampen activin-dependent osteogenic signaling through ACVR1(R206H), particularly under conditions of limiting concentrations of activin ligands or type II BMP receptor binding partners. An alternative, but not mutually exclusive, possibility is that signaling complexes lacking wild-type ACVR1 exhibit greatly enhanced signaling properties, although this is not supported by cell culture experiments with embryonic stem cells^8^ or our preliminary observations. We are also exploring possible indirect effects that could result in enhanced HO at endpoint, including increased FAP proliferation or delayed differentiation.

Skeletal muscle exhibits a robust regenerative capacity, owing to the myogenic activity of satellite cells^45,46^. We showed that in FOP mice, this regenerative response is subverted and the developmental trajectory of injured muscle is instead dominated by endochondral ossification. As FAPs are positive regulators of muscle regeneration under normal physiological conditions^13,16,17,47,48^, it is reasonable to hypothesize that pro-myogenic functions of FAPs are directly or indirectly disrupted by expression of ACVR1(R206H) in the presence of activin ligands. Whereas the histological appearance of muscle from wild-type and FOP mice is similar for approximately 3 days post-injury, the rapid accumulation of regenerated muscle fibers that occurs in wild-type muscle on subsequent days is not observed in FOP muscle, where cartilage accumulations become the dominant histological feature by day 6. These early, profound inhibitory effects on muscle regeneration would predict that therapeutic intervention prior to the onset of cartilage differentiation would be most efficacious, and highlight the importance of evaluating therapeutic treatments, such as activin inhibitors^8^ (present study), retinoic acid receptor-γ agonists^22^, and small molecule ACVR1 (ALK2) inhibitors^19^, for their effectiveness in retaining or restoring muscle regenerative capacity, in addition to inhibition of HO.

## Materials and methods

### Generation of *Acvr1*^*tnR206H*^ knockin mice

CAG-tdTomato-T2A-Neo stop cassette, CT2ANpA, was designed to express both tdTomato red fluorescent protein and a neomycin selection marker under a CAG promoter^49^, and to stop read-through transcription. A self-cleaving 2A peptide-mediated tdTomato-Neo bicistronic construct was generated by replacing the mCherry gene in mCherry-T2A-Neo/BSPKS with the tdTomato gene from EF1pi/tdTom.6× (both kindly provided by Dr. Alex Lichtler). A CAG promoter and three-tandem SV40 polyadenylation sequences were introduced at the 5′ and 3′ ends of tdTomato-T2A-Neo sequence, respectively. The floxed CT2ANpA cassette was generated by introducing the CT2ApA sequence between two loxP sequences. The pPGKDTA_mACVR1 plasmid was generated by subcloning 8.5 kb of mouse *Acvr1* genomic DNA that spans introns 4 to 6 into pFLCI_DTA plasmid^25^, using standard recombineering techniques^50^. To introduce the R206H mutation, site-directed mutagenesis was performed on a plasmid harboring the AflII-XhoI fragment containing exon 5 with the following oligomers; 5′-GTACAGAGAACGGTGGCcCatCAGATAACCCTGTTGGAG-3′and 5′-CTCCAACAGGGTTATCTGatGgGCCACCGTTCTCTGTAC-3′ (the letters shown in lowercase are mutated nucleotides). To generate the final targeting vector, DFOP-LTN, wild-type exon 5 was replaced with the mutated exon and the floxed CT2ANpA fragment was inserted into the NdeI site in intron 4 of pPGKDTA_mACVR1. The detailed cloning strategy and complete sequence of plasmids are available by request.

ES cell electroporation and production of chimeric mice were performed by the University of Connecticut Gene Targeting and Transgenic Facility (GTTF). The DFOP-LTN plasmid was linearized with AclI and electroporated into 129S6/C57BL/6 hybrid ES cells (D1: established by GTTF). Nested polymerase chain reaction (PCR) was used to screen for homologous recombination on both 5′ and 3′ ends with the following primers; 5′ end: 1st PCR, 5′-GAGGCGGCTCCGAGGGTAAAGATG-3′ and 5′-GCTCACCTCGACCATGGTAATAGCG-3′, 2nd PCR, 5′-AACTCCCACAAGCTGTCCTCTGAC-3′ and 5′-CCGTAAGTTATGTAACGCGGAACTCC-3′; 3′ end: 1st PCR, 5′-GGCTCGACCTCGACCGGGATAAC-3′ and 5′-CAAGCAGAACCACCCAAGGAGCC-3′, 2nd PCR, 5′-TGCTATACGAAGTTATAGATCTATGG-3′ and 5′-GTGAACAACGGAGCAGAGCAGGG-3′. The targeted allele generated 2.0 and 6.7 kb diagnostic products with the 5′ and 3′ nested PCR reactions, respectively. The presence of the R206H-causing mutation was confirmed by generation of a diagnostic 203 bp fragment with the following primers: 5′- CAACAGGGTTATCTGATGG-3′ and 5′- TTGGAGTTGCTCTCAGGAA-3′. Chimeric mice were produced from three targeted ES clones by aggregation with CD1 embryos. Germ line transmission of the targeted allele was assessed by the presence of red fluorescence, PCR for the R206H-causing mutation, and the 5′ and 3′ nested PCR reactions. Two *Acvr1*^*tnFOP*^ knockin lines were established, one of which is described herein.

### Mice crosses and genotyping

Animal procedures were reviewed and approved by the University of Connecticut’s Institutional Animal Care and Use Committee. Tie2-Cre transgenic mice^10^ were kindly provided by Dr. Tom Sato (UT Southwestern). VE-Cadherin-Cre transgenic mice^27^ (B6.Cg-Tg(Cdh5-cre)7Mlia/J), Pdgfrα-Cre transgenic mice^33^ (Tg(Pdgfra-cre)1Clc) and SCID mice (B6.CB17-Prkdc^scid^/SzJ) were obtained from Jackson Laboratories (USA). *R26*^*NG*^ Cre-dependent GFP reporter mice^25^, *Alk2*^*flox*^ conditional knockout mice^35,36^ (referred to here as *Acvr1*^*flox*^) and *MyoD*^*iCre*^ knockin mice^24^ have been described previously. To induce cell-specific recombination of the *Acvr1*^*tnR206H*^ allele, *Acvr1*^*tnR206H/+*^ mice were first mated with *R26*^*NG/+*^ mice. The resulting *Acvr1*^*tnR206H/+*^;*R26*^*NG/+*^ females were then mated with Cre expressing males to obtain experimental mice. *Acvr1*^*tnR206H/+*^;*R26*^*NG/+*^;Tie2-Cre males were mated with *Acvr1*^*flox/flox*^ females to obtain *Acvr1*^*tnR206H/flox*^;*R26*^*NG/+*^;Tie2-Cre mice. Except for SCID mice, all mice were maintained on an FVB-enriched background. Experimental mice were adults between 8 and 16-weeks-of-age, except where noted. As sex-specific differences in the progression of HO were not observed, male and female mice were used interchangeably in all studies.

Experimental mice were genotyped by PCR and reporter fluorescence. The following primers were used for genotyping: *Acvr1*: 5′-TGCTGTCTTTTAACTCCTGGGATC-3′ and 5′-AGTACTCTTGTGTGTGTGCTTATG-3′ *Acvr1*^*tnR206H*^: 5′-CAACAGGGTTATCTGATGG-3′ and 5′-TCACATGTCCAGAGTTGCT-3′; *Acvr1*^*flox*^: 5′-TGCTGTCTTTTAACTCCTGGGATC-3′ and 5′-TCTAAGAGCCATGACAGAGGTTG-3′; *MyoD*^*iCre*^: 5′-CTGGACCCAGGAACTGGAAGCTTG-3′ and 5′-AGCATCTTCCAGGTGTGTTCAGAG; Tie2-Cre: 5′-CCCTGTGCTCAGACAGAAATGAGA-3′ and 5′-CGCATAACCAGTGAAACAGCATTGC-3′; VE-Cadherin-Cre: 5′-CATTTGGGCCAGCTAAACAT-3′ and 5′-CGGATCATCAGCTACACCAG-3′; Pdgfrα-Cre: 5′-GCGGTCTGGCAGTAAAAACTATC-3′ and 5′-GTGAAACAGCATTGCTGTCACTT-3′ and *R26*^*NG*^: 5′-GATCAGCAGCCTCTGTTCCACA-3′ and 5′-CGCTGAACTTGTGGCCGTTTAC-3′. Tissue-specific recombination was visually confirmed by reporter fluorescence at the time of tissue harvest. Mouse colonies were managed using SoftMouse Colony Management software (softmouse.net).

### FACS isolation

FAPs were isolated by FACS as described^34^, with minor modifications, as follows. Total hindlimb muscle was harvested, minced with scissors for 7–9 min and then incubated for 60–75 min at 37 °C in Dulbecco’s Modified Eagle Medium (DMEM) (Life Technologies) supplemented with 700–800 U/ml Collagenase Type II (Worthington Biochemical) and 0.3 U/ml Dispase (Invitrogen) with gentle agitation every 15 min. Enzymatic digestion was then quenched by addition of DMEM containing 20% HyClone™ characterized fetal bovine serum (FBS) (GE Healthcare; Lot# A00168), followed by serial filtration of the cell suspension through 100 µm and 70-µm cell strainers (Falcon). The cell suspension was then centrifuged at 800×*g* for 5 min and resuspended in 10% FBS in 1× Dulbecco’s phosphate-buffered saline (DPBS) (Gibco) for antibody staining. Anti-CD31 (1:100, Miltenyi Biotech, #130-097-418) and anti-CD45 (1:100, Miltenyi Biotech, #130-052-301) microbead conjugated antibodies were used to deplete endothelial and hematopoietic cells, respectively, via MACS LS Separation columns (Miltenyi Biotech) loaded in a QuadroMACS Separator (Miltenyi Biotech), according to manufacturer’s instructions. Following magnetic depletion, the remaining cell suspension was incubated with fluorescently conjugated SCA-1-V450 (1:400, BD Horizon, #560653) and PDGFRα-APC (1:100, eBioscience, #17-1401-81) antibodies for 30 min on ice, centrifuged at 800×*g* for 5 min, and resuspended in 2% FBS in DPBS following two washes with 1× DPBS. Samples were filtered through 35-µm cell strainers (Falcon) and 50 µg/mL 7-AAD (BioLegend) was added to a final concentration of 0.50 µg/mL immediately prior to sorting to distinquish between live/dead cells. Fluorescence-minus-one (FMO) controls were used to test for non-specific staining and generate strict sort gates to minimize cross contamination between populations. FAPs were isolated based on co-staining for PDGFRα and SCA-1 as shown in Supplementary Fig. 2. To distinguish between *Acvr1*^*R206H/+*^ and wild-type FAPs, we assessed the enriched hindlimb PDGFRα+ SCA1+ subpopulation for tdTomato and GFP expression, which provides a direct readout of recombination status at the *Acvr1*^*R206H*^ and *R26*^*NG*^ loci, respectively. For the tdTomato-FMOs, depending on the experimental mouse line being analyzed, either *R26*^*NG/+*^*;*Tie2-Cre or *R26*^*NG/+*^*;*Pdgfrα-Cre mice were utilized. *Acvr1*^*tnR206H/+*^ mice were used for the GFP FMOs. Sorting and analysis was done on a FACS Aria II (BD Biosciences) equipped with 407, 488, and 633 lasers. *Acvr1*^*R206H*^ and *R26*^*NG*^ gates were confirmed based on verification of tdTomato and GFP expression via fluorescence microscopy.

### FAP cell culture and expansion

FACS-isolated FAPs were grown on tissue culture plastic in Growth Media: 20% HyClone™ FBS, characterized (GE Healthcare; Lot# A00168) in DMEM (Life Technologies) with 50 U/mL Penicillin, and 50 μg/mL Streptomycin (Pen/Strep; Gibco). FAPs were incubated at 37 ^o^C in a humidified atmosphere at 5% CO_2_, and media was changed every other day. FAPs used in experiments were passaged three or fewer times.

### Transplantation

Following FACS isolation and expansion (described above) FAPs were detached and resuspended in ice-cold phospahte-buffered saline (PBS) at a density of 2 × 10^7^ cells/mL and injected into the gastrocnemius muscle at 1 × 10^6^ cells per 50 µL injection. SCID mice were used as hosts to avoid immunological rejection of donor cells, which were of a mixed genetic background. SCID hosts were pre-injured with 100 µL of 10 μM cardiotoxin (Sigma) 1 day prior to transplantation.

### Ligands and anti-activin antibody

rhBMP2 and activin ligands were obtained from R&D Systems (Minneapolis, MN). A human monoclonal antibody against human activin A (ActA-mAb) and an IgG2a isotype control were provided by Acceleron Pharma (Cambridge, MA). ActA-mAb was produced using the sequence in Amgen patent WO 2008/031061, clone A2.

### Osteogenic assay

FACS-isolated FAPs were expanded in culture as described above, passed at 3 × 10^4^ cells/cm^2^ and grown in Growth Media for 1 additional day. Cells were then grown in media containing 5% FBS/DMEM/Pen/Strep for 8 days, with or without BMP2, activin ligands and ActA-mAb at the indicated concentrations. To assess osteogenic differentiation, cells were rinsed 2× in PBS and fixed in 2–4% paraformaldehyde. ALP activity was detected using a commercial kit according to the manufacturer’s recommendations (Sigma).

### Histology

Tissues were dissected from adult mice and immediately fixed in 4% paraformaldehyde, decalcified in 12% EDTA (pH 7.2), and processed for paraffin embedding. Histological analysis was performed on 9-μm sections. Sections were de-paraffinized, rehydrated, and stained with Alcian Blue and Fast Red, as described^51^.

### Immunohistochemistry

Sections were de-paraffinized, rehydrated, and incubated in 1% hydrogen peroxide with 30% methanol to quench endogenous peroxidases. After blocking with 10% normal goat serum or 1.5% non-fat dried milk for 1 h at room temperature, slides were incubated with primary antibodies in blocking buffer at 4 °C overnight. Rabbit anti-ACVR1 (Abcam; #ab60157) and rabbit anti-SOX9 (Millipore; #AB5535) were used at dilutions of 1:100 and 1:1000, respectively. Sections were washed three times with PBS containing 0.1% Tween-20 and then incubated with the appropriate biotinylated secondary antibody at a dilution of 1:200 (Vector Laboratories). Detection was via Vectastain Elite ABC Reagent (Vector Laboratories) using DAB reagent (Vector Laboratories) followed by counterstaining with Hematoxylin. For negative controls, the primary antibody was omitted. Negative controls did not show staining.

### Immunofluorescence

Frozen sections of 9-μm were rehydrated in PBS, blocked in 1% BSA (Sigma), 10% goat serum (Sigma), and 0.1% Tween 20 in PBS (dilution buffer), and stained with primary antibody in dilution buffer overnight at 4 °C. Rabbit anti-OSX (Abcam; #ab94744) and rabbit anti-SOX9 (Millipore; #AB5535) were used at dilutions of 1:100 and 1:1000, respectively. Sections were then washed in PBS, stained with Alexa Fluor–conjugated secondary antibody at 1:200 in dilution buffer at room temperature for 1 h, washed in PBS, counterstained with DAPI, and coverslipped using Fluoro-Gel (Electron Microscopy Sciences). Controls lacking primary antibody were conducted on adjacent sections simultaneously and did not show staining.

### Imaging

Stained tissues or cells were imaged on a Nikon E600 microscope equipped with a Spot RT3 camera and Spot Advanced image capture software (Diagnostic Instruments) or on a Nikon TE2000-U inverted microscope equipped with a Qimaging Retiga EXi camera and Openlab imaging software (Improvision). A Nikon A1R four-laser spectral confocal microscope (excitation wavelengths of 405, 488, 561, and 640 nm) was used for immunofluorescence microscopy, and images were acquired using Nikon Elements software. Image processing was performed using Nikon Elements software, the GNU Image Manipulation Program, ImageJ, or Photoshop (Adobe). Only minor linear adjustments were made to image brightness and contrast. Images were assembled in Photoshop.

### Western blots

Near-confluent FAP cultures in 6-well plates were serum starved for 2 h and then incubated for 1 h with or without ligand and ActA-mAb at the indicated concentrations. Cells were lysed in RIPA buffer with protease and phosphatase inhibitors (Pierce) by vortexing for 30 min. Protein extracts were diluted two-fold in Laemmli buffer and heated for 10 min at 95 °C prior to SDS–PAGE and subsequent electrophoretic transfer to a PVDF membrane. After 1 h of blocking in 5% non-fat dry milk in Tris-buffered saline (TBS), the membrane was incubated at 4 °C overnight in primary antibody. The membrane was then washed thoroughly with TBS containing 0.1% Tween 20 (TBS-T), incubated for 1 h in secondary antibody, and thoroughly washed again with TBS-T before imaging using the Odyssey^®^ CLx (LI-COR). Primary antibodies used include rabbit anti-phospho SMAD1/5/8 (Cell Signaling Technology; #13820) at 1:750 and mouse anti-β-actin (Cell Signaling Technology; #3700) at 1:2,500. Secondary antibodies included IRDye^®^ 800CW goat anti-rabbit (LI-COR) at 1:7,500 and IRDye^®^ 680RD goat anti-mouse (LI-COR) at 1:10,000. Uncropped Western blots are shown in Supplementary Fig. 11.

### Muscle injury models

The gastrocnemius muscle of adult mice was injured either by forceps pinch or by cardiotoxin injection. For pinch injury, the gastrocnemius and covering skin was gripped at the approximate midbelly of the muscle with 2-mm wide tissue forceps and pressure was applied for 5 s. Care was taken to avoid breaking the skin and incidental contact with the tibia and fibula; the latter was verified by µCT. In subsequent testing with a Randall Selitto Paw Pressure Test Apparatus (IITC Life Science), similarly sized lesions were observed following application of 2200–2700 g of force to the gastrocnemius muscle. For cardiotoxin injury, 100 µL of 10 μM cardiotoxin (Sigma) in PBS was injected into the midbelly of the muscle. Injuries were performed while mice were under isoflurane anesthesia.

### Intramuscular activin A and BMP2 injection

Fifty microliters of 1% methylcellulose (Sigma) in PBS with or without 5 µg of activin A or 2.5 µg BMP2 was injected into the tibialis anterior muscle of adult mice under isoflurane anesthesia.

### Alcian Blue and Alizarin Red skeletal preparations

Mice were fixed in 95% ethanol, defatted in 100% acetone, and stained as whole mounts with 0.03% Alcian Blue 8GX (Acros Organics) in 95% ethanol containing 3% acetic acid to detect cartilage matrix proteoglycans. After rinsing in 95% ethanol, mice were stained with Alizarin Red (Sigma) at 25 mg/L in 1% potassium hydroxide to detect mineralized matrix. Mice were then cleared in 2% potassium hydroxide and stored in 80% glycerol in 20% PBS.

### Antibody treatment

To test effects of ActA-mAb on spontaneous HO, *Acvr1*^*tnR206H/+*^;*R26*^*NG/+*^*;*Pdgfrα-Cre mice were injected subcutaneously with either ActA-mAb or an IgG2a isotype control antibody (both at 10 mg/kg) twice weekly starting at 2-weeks-of-age, for a duration 4 weeks. All mice were scanned by µCT prior to initiation of treatment and mice that exhibited HO were omitted from the study. To test effects of ActA-mAb on injury-induced HO, adult mice were treated twice weekly (10 mg/kg) for the duration of the experiment, beginning on the day of injury (day 0) until endpoint. In some cases, a single antibody dose was given at day 0 or day 3 post-injury, as specified. For transplantation studies, SCID hosts were given a single antibody dose at the time of injury.

### µCT and HO quantification

µCT imaging was performed at enrollment and at endpoint using an IVIS SpectrumCT model 128201 (Perkin-Elmer). All µCT images were taken using the Standard-One Mouse CT acquisition mode for whole body imaging (150 µm voxel size; estimated radiation dose of 52.8 mGy; 140 s scan time) or medium resolution acquisition mode for limbs (75 µm voxel size; estimated radiation dose of 132 mGy; 210 s scan time). Mice were imaged under isoflurane anesthesia. The µCT images were generated and HO volumes were quantified using 3D Slicer software (http://www.slicer.org).

### Statistical analysis

Statistical analysis was performed using GraphPad Prism (GraphPad Software). All numerical values are presented as mean values ± the standard error of the mean (SEM). The Mann–Whitney U test was used to determine significance. No randomization was used to allocate animals to particular groups, and the investigators were not blinded to experimental groups during analysis. The number of animals required to assess statistical significance of a pre-specified effect was estimated based on our prior experience with the models employed.

### Data availability

The authors declare that the main data supporting the findings of this study are available within the article and its Supplementary Information files.

## Electronic supplementary material

Supplementary Information
