# Supplementary material for: Activin-dependent signaling in fibro/adipogenic progenitors causes fibrodysplasia ossificans progressiva
Source: Nat Commun. 2018 Feb 2;9:471. doi: 10.1038/s41467-018-02872-2 (PMC5797136; doi:10.1038/s41467-018-02872-2)
Supplement: Supplementary file 1 — Supplementary Information [file 41467_2018_2872_MOESM1_ESM.pdf]

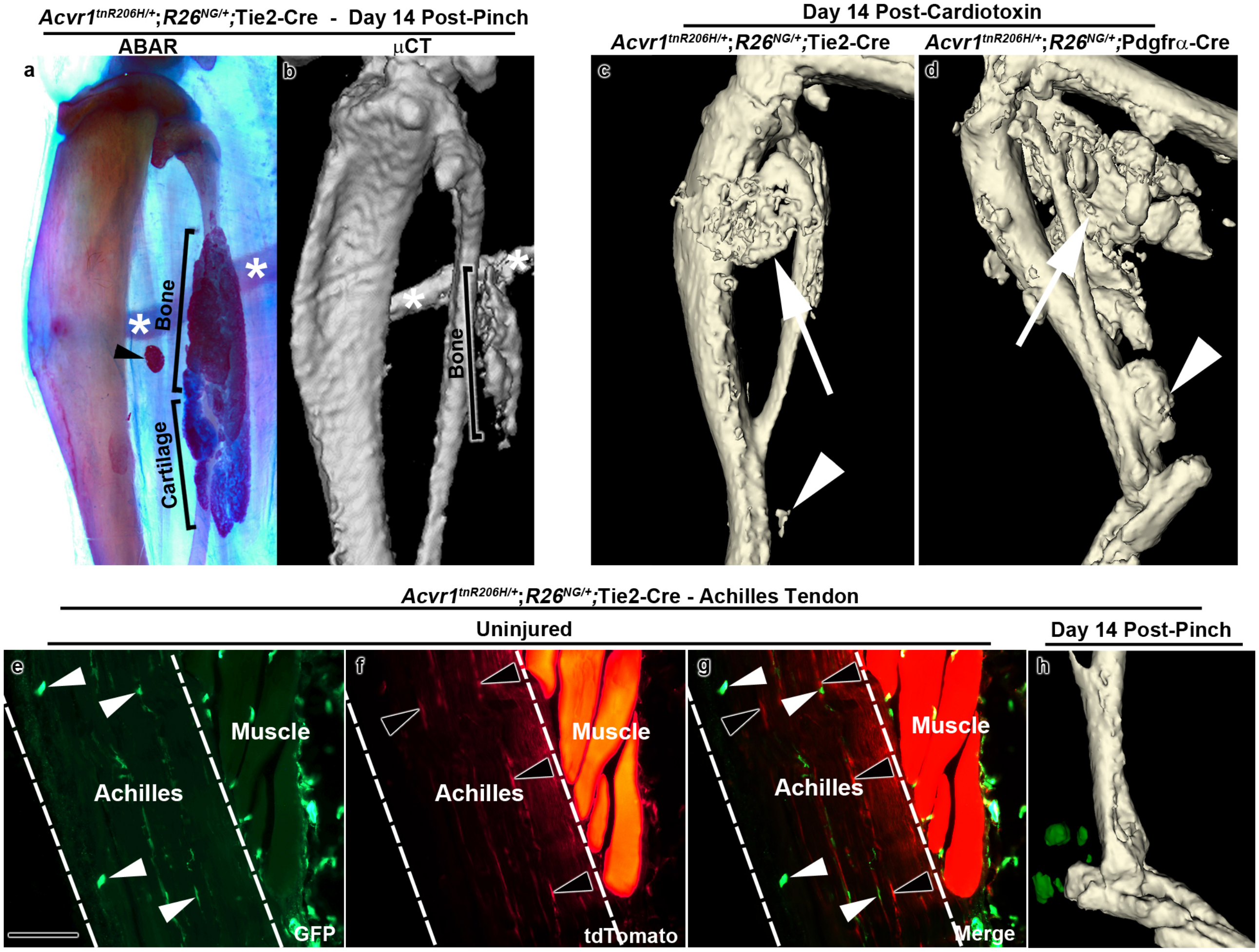

Supplementary Figure 1. Comparison of visualization methods, mode of injury, and extent of HO in two FOP models. (a, b) Whole-mount ABAR and μCT images of the same distal hindlimb of an *Acvr1*$^{tnR206H/+}$;*R26*$^{NG/+}$;Tie2-Cre mouse 14 days after pinch injury. ABAR staining (a) permits visualization of cartilaginous (blue, bracket) and boney (red, bracket and asterisks) components of HO lesions, whereas only mineralized bone is observed by μCT (b). ABAR staining also permits observation of heterotopic bone that is insufficiently mineralized for detection by μCT (a, arrowhead). (c, d) Examples of HO following cardiotoxin-induced muscle injury in *Acvr1*$^{tnR206H/+}$;*R26*$^{NG/+}$;Tie2-Cre (c; n=7 mice) and *Acvr1*$^{tnR206H/+}$;*R26*$^{NG/+}$;Pdgfrα-Cre (d; n=7 mice) mice. Both models exhibit intramuscular (arrows) and intratendinous (arrowheads) HO. (e-g) A frozen section of the Achilles tendon (boundry marked by white dotted lines) and associated hindlimb muscle of an *Acvr1*$^{tnR206H/+}$;*R26*$^{NG/+}$;Tie2-Cre mouse (n=2 mice). (e) GFP+ lineage-marked cells are detected within the Achilles tendon (white arrowheads). (f) tdTomato+, unrecombined cells are also present within the Achilles tendon (black arrowheads). (g) Merge of panels e, f. (h) Pinch injury of the Achilles tendon of an *Acvr1*$^{tnR206H/+}$;*R26*$^{NG/+}$;Tie2-Cre mouse resulted in HO at 14 days post-injury (n=3 mice). Scale bar = 50 μm (e-g).

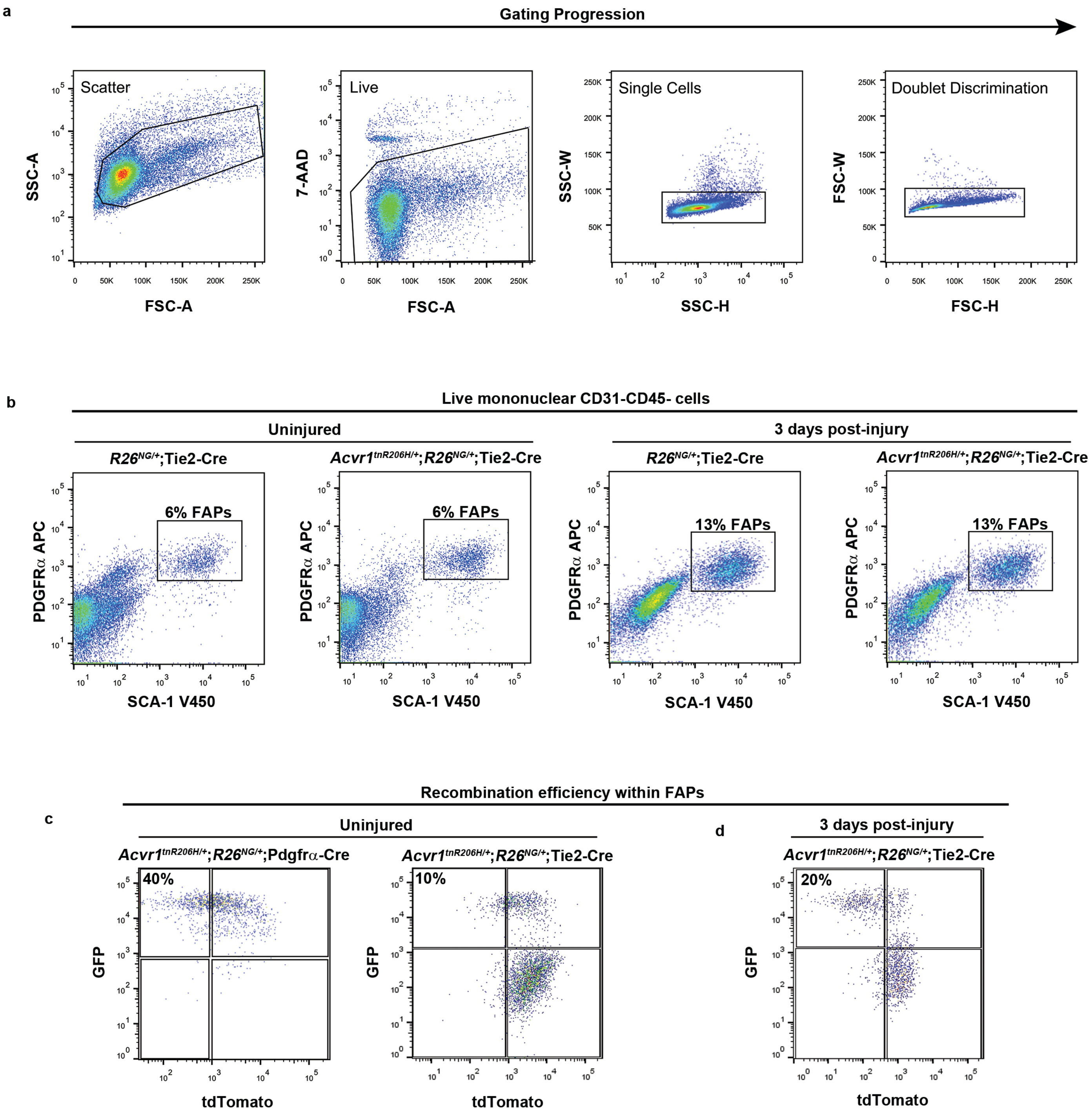

Supplementary Figure 2. FACS gating strategy for isolation of *Acvr1*$^{R206H/+}$ FAPs and analysis of recombination efficiency. Plots shown are representative of the cell analyses and sorting data presented herein (n=5 mice per genotype). The total mononuclear cell pool was magnetically depleted for CD31+ and CD45+ cells prior to FACS. (a) CD45-CD31- mononuclear cells were gated for forward/side scatter to eliminate debris, live/dead, and doublet discrimination. (b) FAPs were identified as the PDGFRα+SCA1+ fraction. FAPs (boxes) accounted for a similar percentage of cells in *R26*$^{NG/+}$;Tie2-Cre *and Acvr1*$^{tnR206H/+}$*;R26*$^{NG/+}$;Tie2-Cre mice both in uninjured muscle and at day 3 post-cardiotoxin injury. (c) Recombination efficiency of the *Acvr1*^{tnR206H} and *R26*^{NG} alleles, as reflected by the percentage of GFP+ and tdTomato- FAPs, was greater with the Pdgfrα-Cre driver than with the Tie2-Cre driver. (d) The GFP+tdTomato- FAP fraction increased after injury.

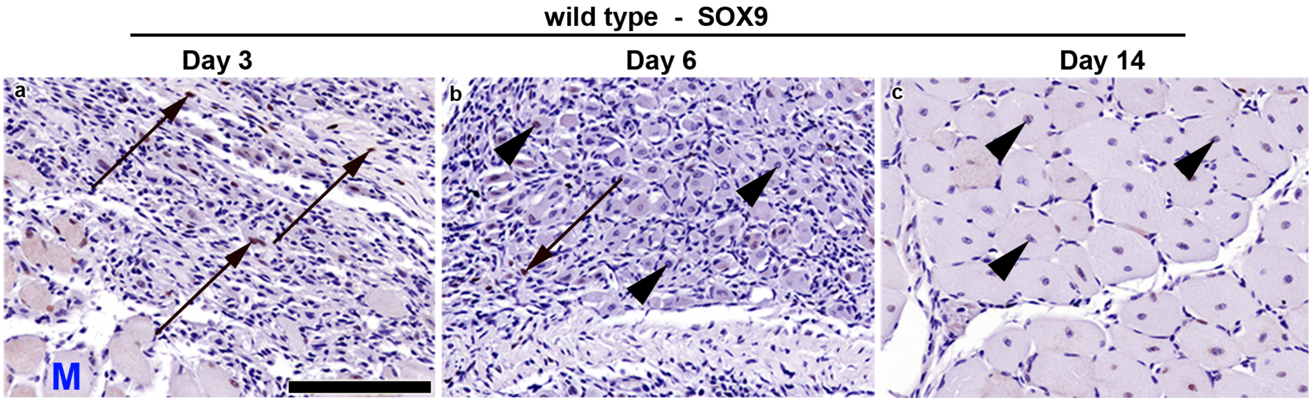

Supplementary Figure 3. IHC analysis of SOX9 expression in wild type pinch-injured muscle. (a-c) Paraffin sections of the gastrocnemius muscle 3, 6, and 14 days after pinch injury (n=5 mice per timepoint). Occasional, SOX9+ cells (brown; examples at arrows) were present at days 3 and 6, but were rare or absent at day 14. Regenerated muscle fibers are identifiable by their central nucleation (examples at arrowheads). Sections are hematoxylin-stained. M, uninjured muscle. Scale bar = 100 µm (a-c).

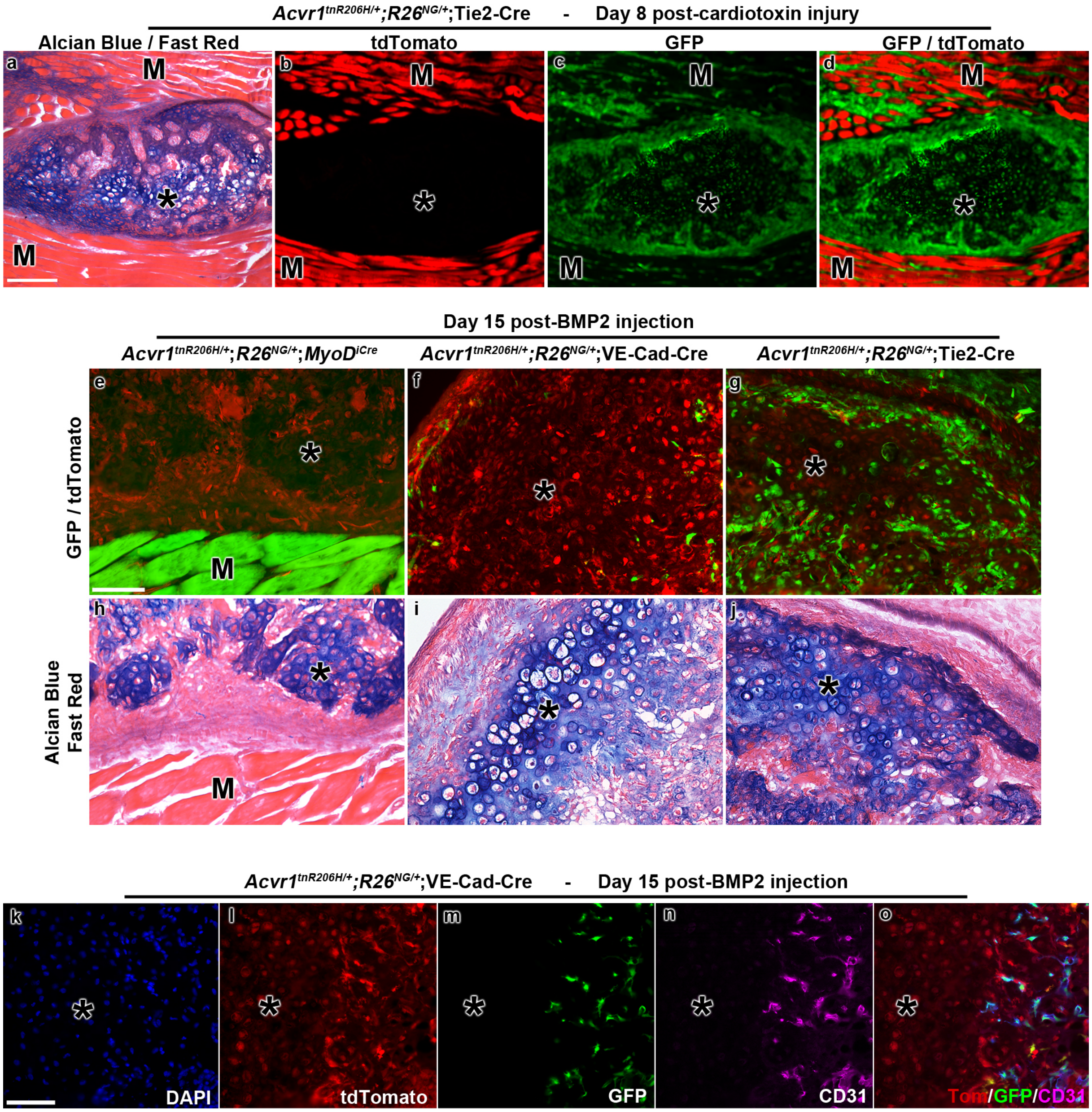

Supplementary Figure 4. *$Acvr1^{R206H}$* functions cell-autonomously in FAP-directed HO, but does not confer osteogenic or chondrogenic capacity to myogenic or endothelial cells in vivo. (a-d) Frozen sections of an *$Acvr1^{tnR206H/+}$*;*$R26^{NG/+}$*;Tie2-Cre lesion (asterisk) embedded in hindlimb muscle (M) 8 days post-cardiotoxin injury (n=4 mice). (a) Alcian Blue / Fast Red staining shows areas of cartilage (blue) and bone. (b) Nearby section showing the absence of tdTomato fluorescence within the lesion and intense tdTomato fluorescence in surrounding muscle. (c) Virtually all lesional skeletal cells are GFP labeled. GFP labeling of skeletal muscle is predominantly vasculature, due to Tie2-Cre expression in endothelium[10,12]. (d) Overlay of GFP and tdTomato fluorescence. (e-g) tdTomato+ cells were readily detected in BMP2-induced skeletal lesions (asterisks), regardless of the Cre driver used. GFP+, lineage marked skeletal lesional cells are exceedingly rare or absent when *$MyoD^{iCre}$* (e; n=3 mice) or VE-Cadherin-Cre (f; n=3 mice) drivers are used, but are abundant when Tie2-Cre drives *$Acvr1^{tnR206H}$* and *$R26^{NG}$* recombination (g; n=4 mice). (h-j) Cryosections of lesional areas close to those in (e-g). Sections were stained with Alcian blue / Fast Red to reveal lesion histology. Asterisk, lesional tissue; M, muscle. (k-o) A frozen section of BMP2-induced lesional tissue embedded within hindlimb muscle of an *$Acvr1^{tnR206H/+}$*;*$R26^{NG/+}$*;VE-Cadherin-Cre mouse. Nuclei in (k) were stained with DAPI. Lesional tissue is comprised of *$Acvr1^{tnR206H}$*-unrecombined cells (l; tdTomato+) except for GFP+ vasculature (m), identified by CD31 immunofluorescence (n). The asterisk identifies a chondrogenic lesional area, which is avascular. (o) Overlay of panels l, m, n. Scale bars = 200 µm (a-d), 80 µm (e-o).

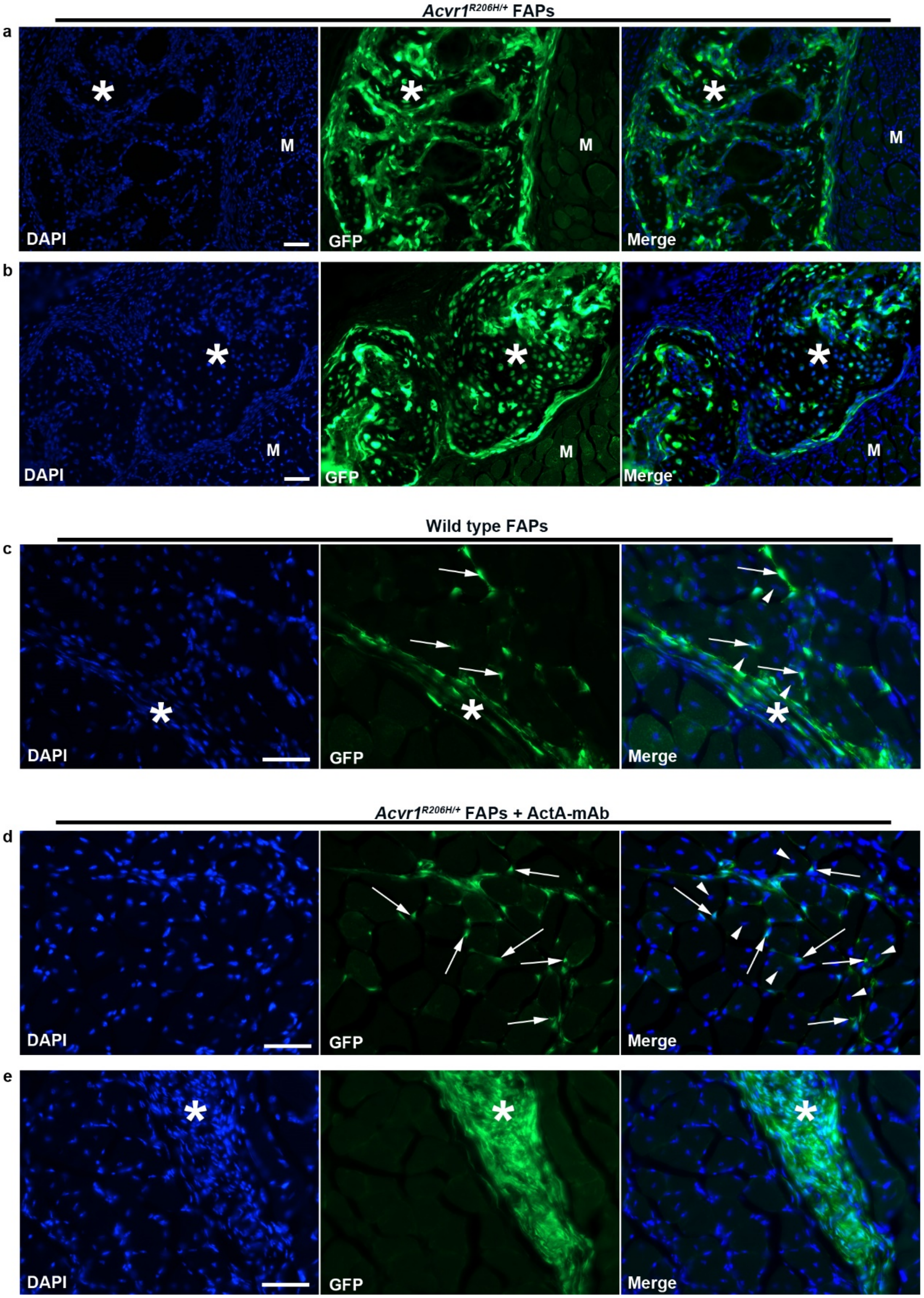

Supplementary Figure 5. Transplanted *Acvr1*$^{R206H/+}$ FAPs exhibit wild type-like behavior under conditions of activin blockade. (a, b) Cryosections of hindlimb muscle 21 days post-transplantation of FACS-isolated *Acvr1*$^{R206H/+}$ FAPs from *Acvr1*$^{tnR206H/+}$;*R26*$^{NG/+}$;Tie2-Cre donors into the pre-injured gastrocnemius muscle of SCID hosts (n=5). GFP+ FAPs gave rise to ectopic bone (a; asterisk) and cartilage (b; asterisk) embedded within the muscle (M). (c) Similarly transplanted FAPs from *R26*$^{NG/+}$;Tie2-Cre mice (wild type for *Acvr1*) established residence in the interstitium (examples of transplanted cells at arrows) among regenerated muscle fibers (arrowheads), or formed aggregates of fibrotic tissue (asterisk) (n=5). (d, e) *Acvr1*$^{R206H/+}$ FAPs transplanted into SCID hosts that received ActA-mAb at the time of injury did not undergo HO, and instead, were frequently observed in the muscle interstitium surrounding regenerated myofibers (d; arrows) or forming aggregates of fibrotic tissue (e; asterisk) (n=5). Scale bars = 100 μm (a-e).

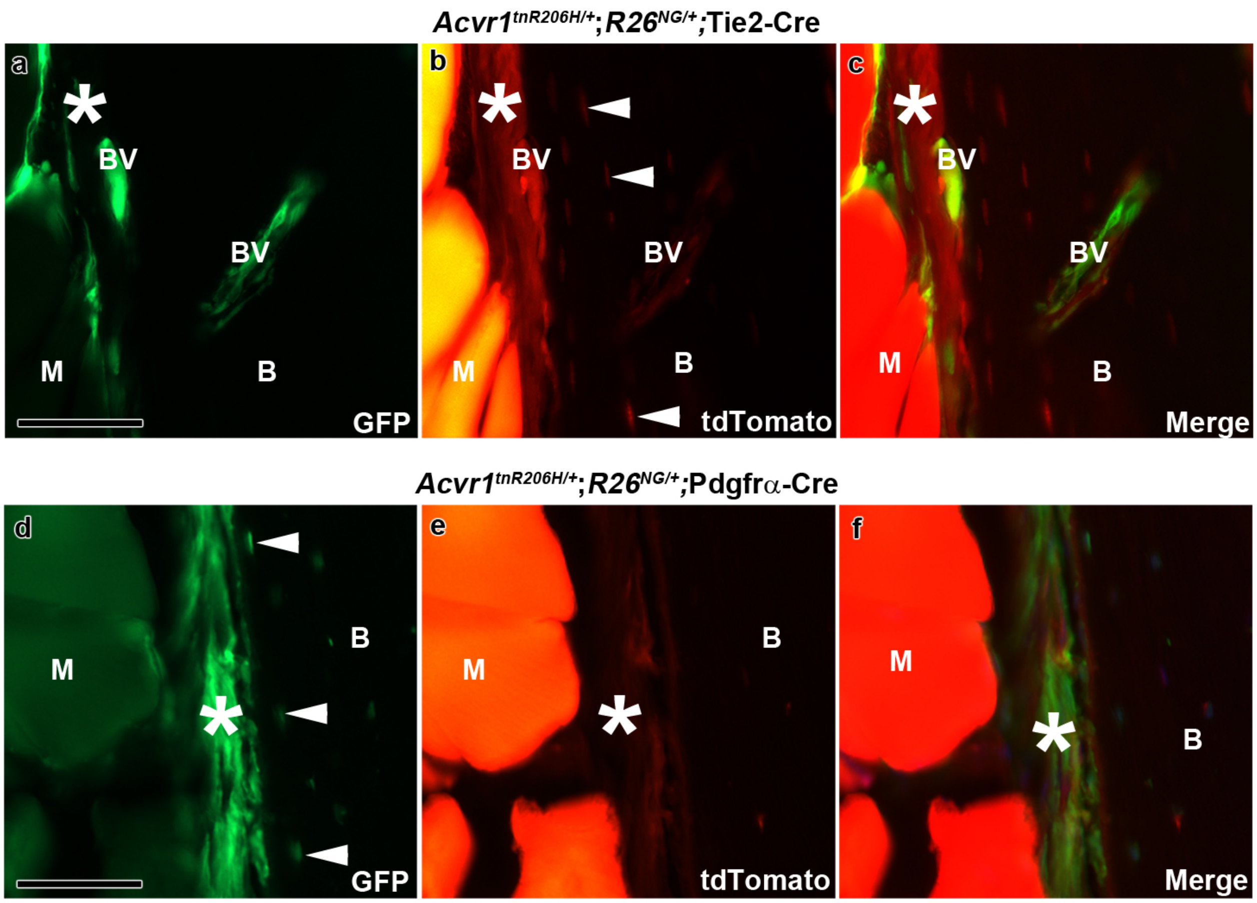

Supplementary Figure 6. Comparison of lineage labeling between Pdgfrα-Cre and Tie2-Cre drivers.(a-c) Cryosection through the hindlimb of an *Acvr1*$^{tnR206H/+}$;*R26*$^{NG/+}$;Tie2-Cre mouse. Blood vessels (BV), but not the periosteum (asterisks) or osteocytes (b; arrowheads) of endogenous bone (B), are lineage labeled with GFP. Cells that are unrecombined at the *Acvr1*^{tnR206H} locus are tdTomato+ (n=4 mice). (d-f) Cryosection through the hindlimb of an *Acvr1*$^{tnR206H/+}$;*R26*$^{NG/+}$;Pdgfrα-Cre mouse. The periosteum (asterisks) and endogenous osteocytes (arrowheads) are lineage labeled (n=5 mice). Scale bars = 50 μm (a-f)

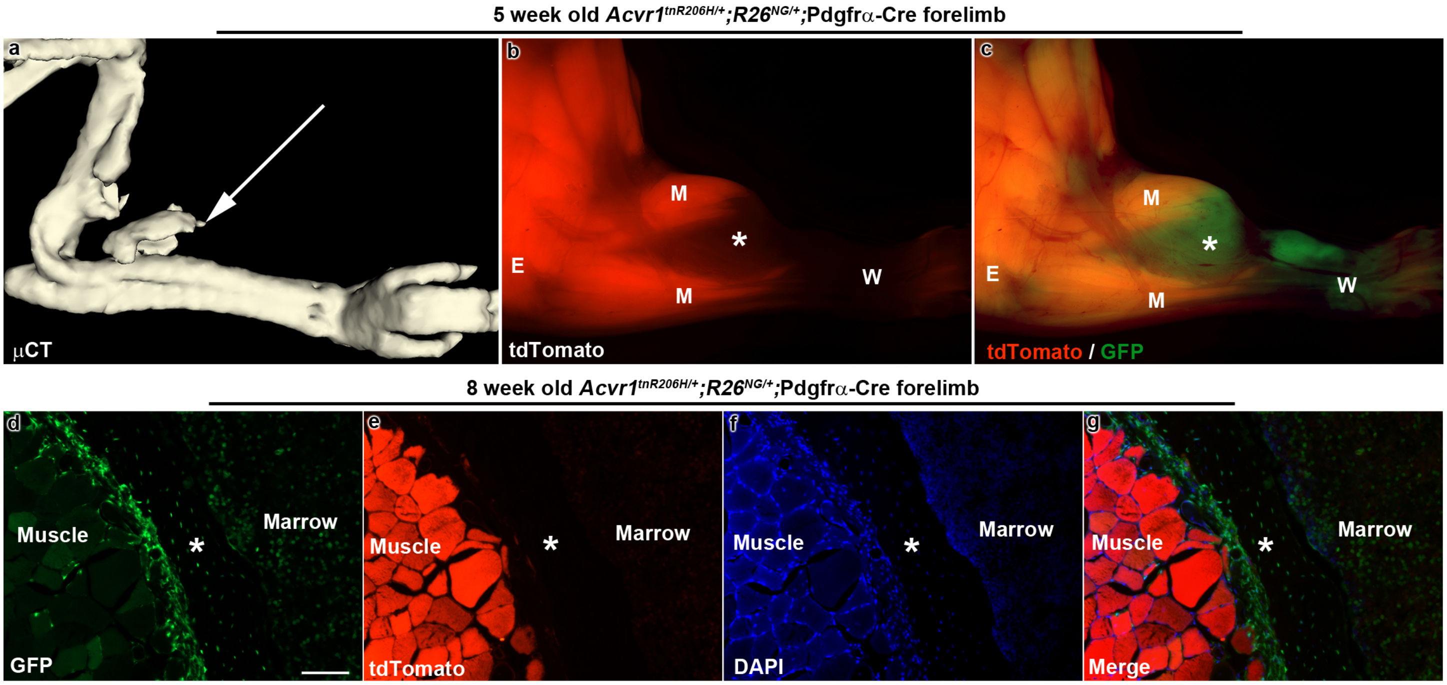

Supplementary Figure 7. The *Acvr1^{R206H}* mutation functions cell-autonomously in FAPs during spontaneous HO. (a) µCT image showing mineralized spontaneous HO (arrow) in a 5-week-old *Acvr1$^{tnR206H/+}$*;*R26$^{NG/+}$*;Pdgfrα-Cre mouse forelimb. (b, c) Whole-mount fluorescence image of the same limb, showing tdTomato+ muscle (M) surrounding the tdTomato-GFP+ lesion (asterisks), which displaced the forearm musculature (M). E, elbow; W, wrist. (d-g) Cryosection of spontaneous HO from the forelimb of an adult *Acvr1$^{tnR206H/+}$*;*R26$^{NG/+}$*;Pdgfrα-Cre mouse (n=7 mice). Cells comprising the heterotopic bone (asterisk) were lineage-labeled (d; GFP+) and recombined at the *Acvr1^{tnR206H}* locus (e; tdTomato-). (g) Overlay of panels d-f. Scale bar = 100 µm (d-g).

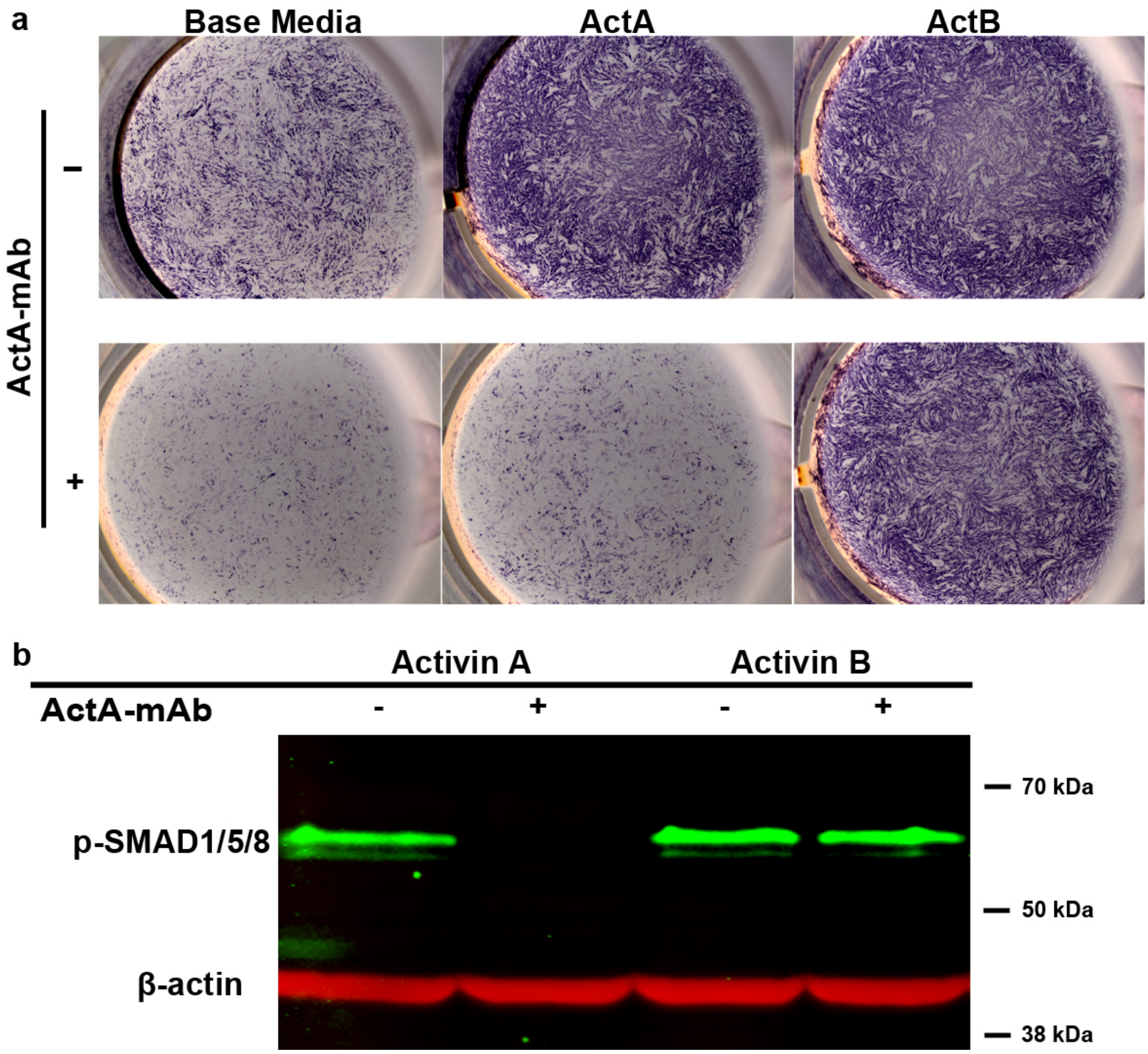

Supplementary Figure 8. In vitro osteogenic response of *Acvr1*$^{R206H/+}$ FAPs to activins A and B and blocking activity of ActA-mAb. FACS-purified *Acvr1*$^{R206H/+}$ FAPs were collected from *Acvr1*$^{tnR206H/+}$;*R26*$^{NG/+}$;Tie2-Cre mice and assayed for osteogenic differentiation by ALP staining (purple). (a) Cells were grown in base media (5% FBS/DMEM), or media + 25 ng/mL (~1 nM dimers) of activin A or B as shown, either with (bottom row) or without (top row) 1μg/mL ActA-mAb (7-fold molar excess). ActA-mAb inhibited the osteogenic response of *Acvr1*$^{R206H/+}$ FAPs to activin A but not activin B. The moderate stimulatory effect of base media alone was also inhibited by ActA-mAb.(b) Western blot of SMAD 1/5/8 phosphorylation. *Acvr1*$^{R206H/+}$ FAPs were serum-starved for 2 hours and SMAD 1/5/8 phosphorylation assayed after a 1-hour incubation in 50 ng/mL activin ligand in DMEM, either with or without co-incubation with 1 μg/mL ActA-mAb. β-actin was used as the loading control. For both (a) and (b), n=3 experiments; representative examples shown.

Acvr1$^{tnR206H/+}$**;*R26***$^{NG/+}$**;Tie2-Cre**

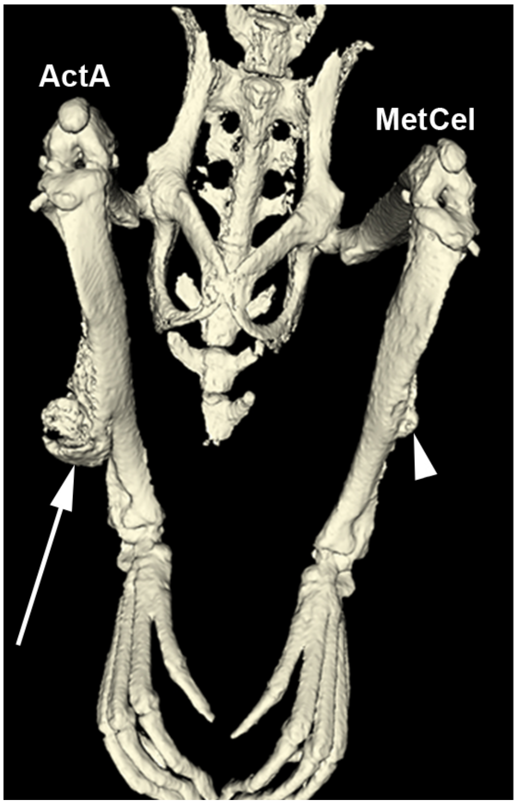

Supplementary Figure 9. Activin A injection may lower the threshold for injury-induced HO in *Acvr1*$^{tnR206H/+}$**;*R26***$^{NG/+}$**;Tie2-Cre mice.** µCT image of an *Acvr1*$^{tnR206H/+}$;*R26*$^{NG/+}$;Tie2-Cre mouse injected with 5 µg activin A (ActA) in 1% methycellulose (MetCel) carrier into the right distal hindlimb and 1% MetCel carrier alone into the contralateral limb (n=2 mice). HO was detected in both limbs, although the extent of HO was greater in the ActA injected limbs (arrows) than in the MetCel injected limbs (arrowheads). The mild injury caused by MetCel injection alone was not sufficient to induce HO in *Acvr1*$^{tnR206H/+}$;*R26*$^{NG/+}$;Tie2-Cre mice that did not receive activin A (see Fig. 4).

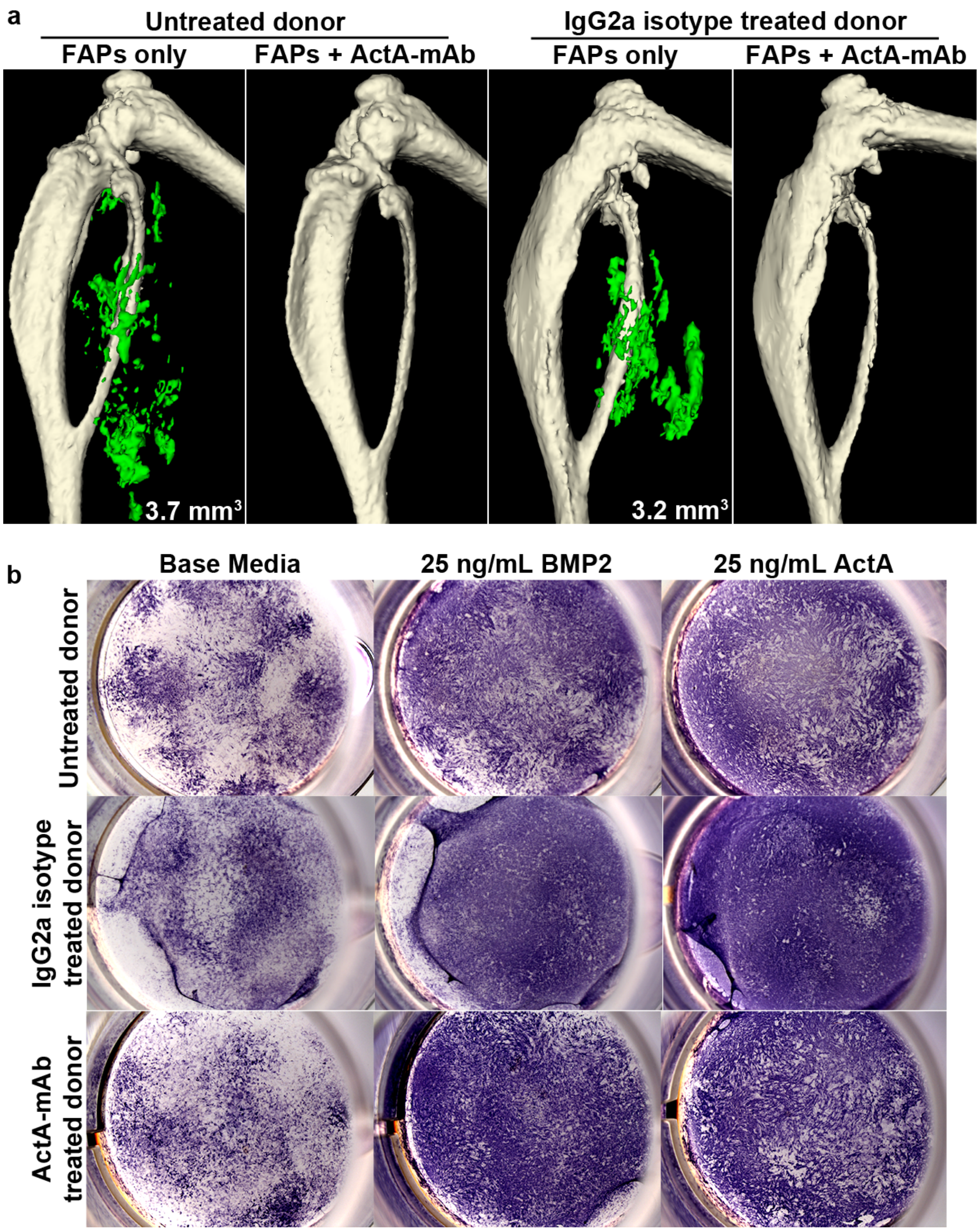

Supplementary Figure 10. Chronic administration of ActA-mAb to donor *Acvr1*$^{tnR206H/+}$;*R26*$^{NG/+}$;Pdgfrα-Cre mice does not alter the osteogenic behavior of donor-derived *Acvr1*$^{R206H/+}$ FAPs. *Acvr1*$^{R206H/+}$ FAPs were collected by FACS from 42-day-old donor mice and tested for osteogenic activity following transplantation and in cell culture (n=3 mice per treatment group). (a) Control experiments in which transplanted *Acvr1*$^{R206H/+}$ FAPs were derived from donor mice that were either untreated or dosed with IgG2a control antibody from 14- to 42-days-of-age. Osteogenic differentiation was quantified by µCT 21 days after transplantation into the pre-injured gastrocnemius muscle of SCID hosts. HO was inhibited when SCID hosts were pre-treated with 1 dose of 10 mg/kg ActA-mAb. HO was pseudocolored green and heterotopic bone volume is given (mm^3). (b) In vitro osteogenic differentiation of donor *Acvr1*$^{R206H/+}$ FAPs, as assayed by ALP staining (purple). Osteogenic differentiation of *Acvr1*$^{R206H/+}$ FAPs derived from ActA-mAb-treated donors was comparable to that of *Acvr1*$^{R206H/+}$ FAPs derived from untreated or IgG2a-treated donors.

a

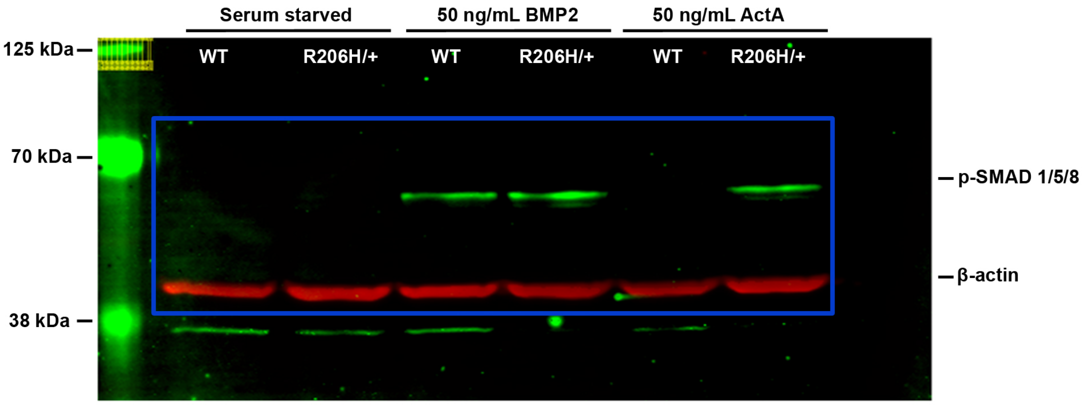

b

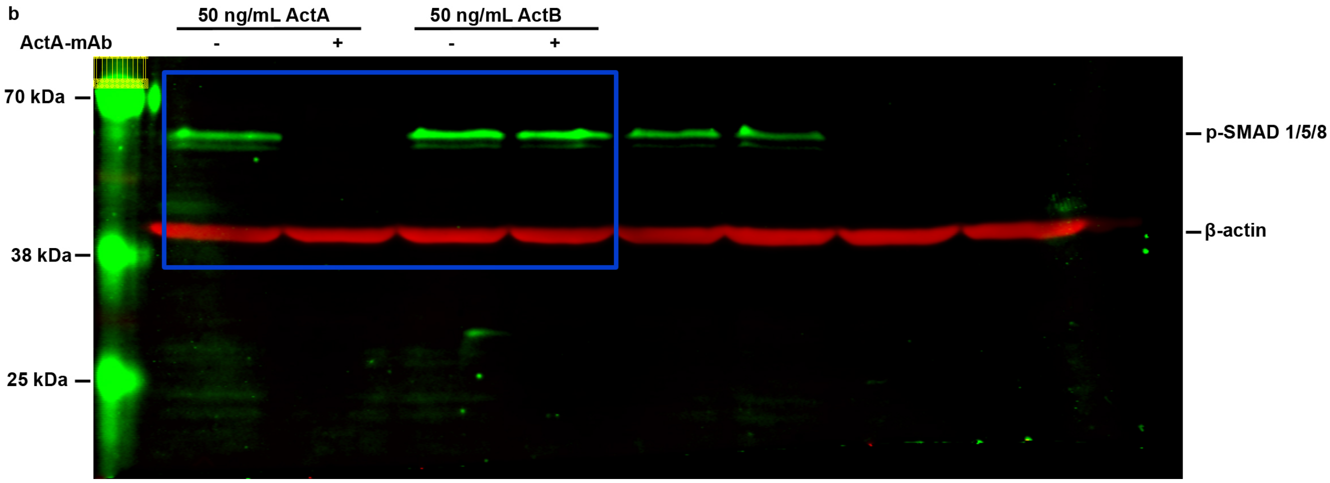

Supplementary Figure 11. (a, b) Uncropped Western blots from Fig. 4b (a) and Supplementary Fig. 8b (b). Cropping is designated by the blue boxes.

Supplementary Table 1

Cre lines	Specificity#	Spontaneous HO	Injury-induced HO
Wild Type	N/A	0/15+	0/25
MyoD^{iCre} [24,27]	Satellite cells, myoblasts, muscle fibers	0/9+	0/12
VE-Cadherin-Cre[28]	Endothelium, hematopoietic progenitors	0/11+	0/15
Tie2-Cre[10]	FAPs[12], endothelium, hematopoietic progenitors	12/15++	42/42
Pdgfrα-Cre[34]	FAPs and periosteum (present study), neuroectoderm, mesenchyme	35/35@	7/7

\# Specificity determined in original cited publications, except where noted.

\+ Mice were analyzed up to 16-months-of-age.

++ Mice were analyzed up to 16-months-of-age. Age-of-onset was from 5.5-9 months-of-age.

@ All mice showed HO by 1-month-of-age. Age-of-onset was from 2-4 weeks-of-age
